# Germinal centers output clonally diverse plasma cell populations expressing high and low affinity antibodies

**DOI:** 10.1016/j.cell.2023.10.022

**Published:** 2023-11-10

**Authors:** Adrien Sprumont, Ana Rodrigues, Simon McGowan, Colin Bannard, Oliver Bannard

**Affiliations:** 1 MRC Human Immunology Unit and MRC Weatherall Institute of Molecular Medicine, University of Oxford, OX3 9DS, UK; 2 Computational Biology Research Group, MRC Weatherall Institute of Molecular Medicine, University of Oxford, OX3 9DS, UK; 3 Department of Linguistics and English Language, University of Manchester, M13 9PL, UK

## Abstract

Germinal centers (GCs) form in lymph nodes after immunisation or infection to facilitate antibody affinity maturation and memory and plasma cell (PC) development. PC differentiation is thought to involve stringent selection for GC B cells expressing the highest affinity antigen receptors, but how this plays out during complex polyclonal responses is unclear. We combine temporal lineage-tracing with antibody characterisation to gain a snapshot of PCs developing during influenza infection. GCs co-mature B cell clones with antibody affinities spanning multiple orders of magnitude, yet each generate PCs with similar efficiency, including weak binders. Within lineages, PC selection is not restricted to variants with highest affinity antibodies. PC differentiation is commonly associated with proliferative expansion to produce “nodes” of identical cells. Immunisation-induced GCs generate fewer PCs but still of low and high antibody affinity. We propose that generating low-affinity antibody PCs reflects an evolutionary compromise to facilitate diverse serum antibody responses.

## Introduction

The generation of antigen-specific antibody responses is a central function of the adaptive immune system that is essential for infection control and long-term immunity. The binding affinity of antibodies improves over the course of immune responses as a consequence of activated B cells collaborating with follicular helper T cells to establish, in secondary lymphoid tissues, specialised structures known as germinal centers (GCs). There, antibody affinity maturation occurs by GC B cells engaging in iterative cycles of somatic hypermutation (SHM) and selection – a form of directed molecular evolution ^1,2^. GC B cells do not themselves secrete antibodies; however, rare differentiation events ensure a continuous low-level output of antibody-secreting plasma cells (PCs) ^3^. Seminal studies utilising clonally-restricted B cell models, tracking B cell receptor (BCR, membrane antibody) knock-in and hapten-specific cells, provided evidence that PC selection is strongly (or almost exclusively) restricted to GC B cells expressing the highest affinity BCRs ^4,5^. This contrasts with memory B cell selection, which preferentially favours cells expressing low affinity BCRs and thereby supports diversity^6,7^.

The clonally-restricted settings used to study PC differentiation differ substantially from typical immune challenges such as vaccination and infection. GC responses against complex pathogen antigens typically involve hundreds of B cell clones recognising multiple overlapping and unique epitopes ^8^. Each lineage diversifies as GC B cells acquire somatic mutations that change the steric interactions and molecular bonds supporting antibody-antigen binding ^1,9^. GCs are considered highly competitive environments, however polyclonal GC responses concurrently support cells with a broad spread of antibody affinities/avidities, including some that are too weak to measure using standard biochemical and FACS assays ^7,8,10^. How affinity-based PC selection plays out in these more complex settings is not clear. Possible scenarios include that only B cells from clonal lineages with the highest affinity BCRs differentiate, that differentiation occurs across lineages but only for the best somatic variants, or that additional immunodominance factors strongly favour certain clones. The outcome will impact the repertoire breadth of serum antibody responses.

Here, we combine genetic fate-mapping ^6^ and single B cell immunoglobulin gene sequencing/cloning ^8,11^ to dissect the development of PCs in polyclonal GCs during influenza infection and after subunit immunisation. We compare the affinities of PC-derived antibodies to those from concurrent GC responses, thereby providing a detailed account of how PC selection plays out in complex polyclonal responses. Our experiments confirm that GCs support the side-by-side maturation of B cells with very different BCR affinities and reveal that cells from across a wide antibody affinity spectrum generate PCs. We propose that this outcome reflects imprecise discrimination for antibody affinity by GC B cells in such settings, with the result being PC populations that approximately mirror the maturation pathways from which they develop. This provides the evolutionary benefit of seeding diverse serum antibody responses.

## Results

### Clonally-restricted GC B cells expressing high affinity antigen receptors preferentially generate plasma cells

We optimised an approach to identify and characterise newly matured PCs using tamoxifen-controlled GC fate-mapping mice (*S1pr2*-CreERT2, Rosa26-LSL-tdTom mice, hereafter termed S1pr2^tdTom^) ^6^. Earlier studies examined the association between antibody affinity and acquisition of a Blimp1^+^ phenotype in clonally-restricted responses ^5^, however GC lineage tracing was not possible then and Blimp1 expression alone may not always mark full PC commitment ^3,12,13^. We therefore revisited this issue using B cells from S1pr2^tdTom^ SW_HEL_ mice to identify newly minted GC-derived PCs ^14^. SW_HEL_ B cells express paired heavy and light chains that bind HEL^3x^ antigen with an equilibrium dissociation constant (K_D_) of ∼100nM ^15,16^, but affinity-mature by acquiring a Y53 mutation that confers ∼100-fold improvement ^5^. This affinity leap can be tracked by staining with fluorescent monomeric HEL^3x^ protein.

**Figure 1:**
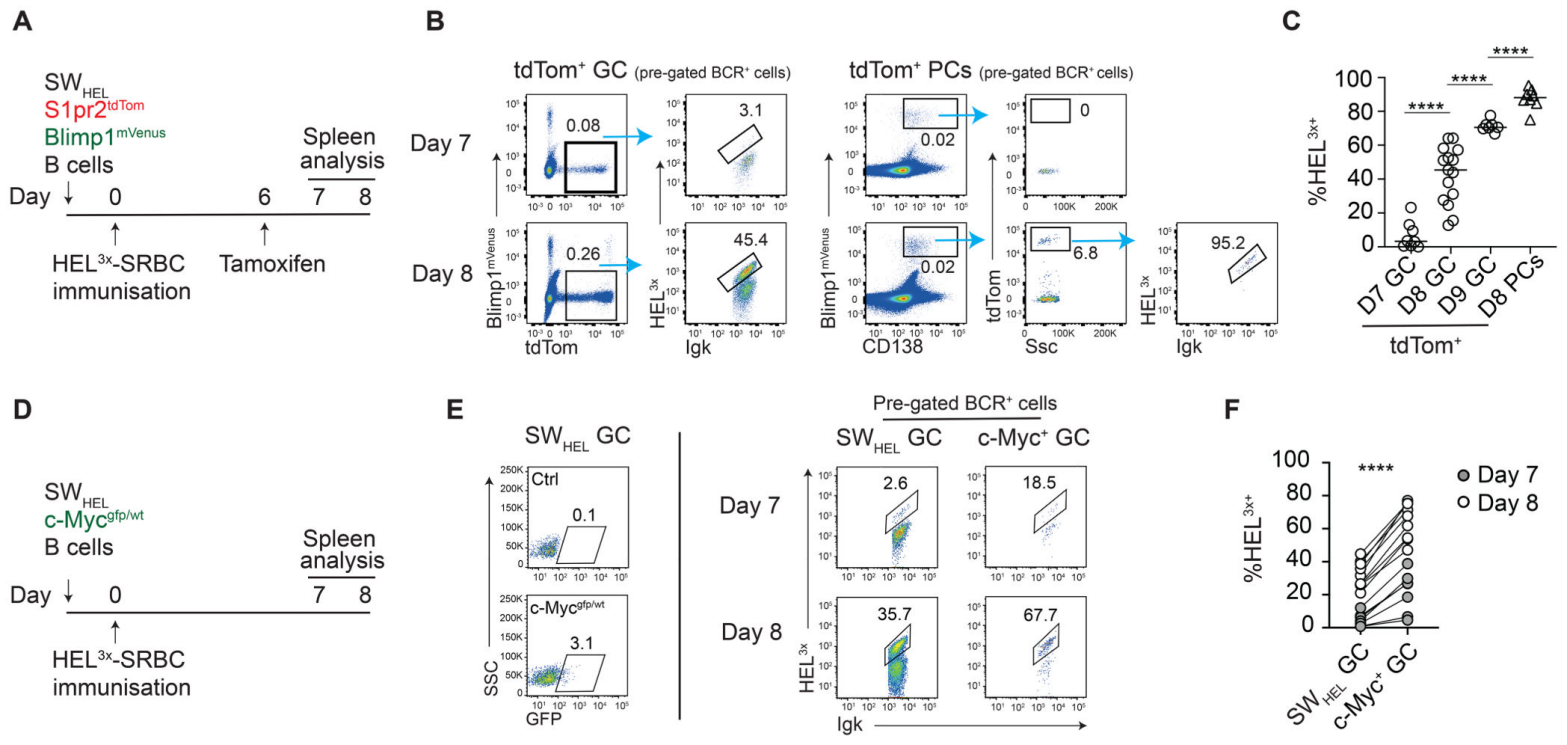
Clonally-restricted GC B cells expressing high affinity BCRs preferentially generate plasma cells A. Experimental scheme for B-C. B. FACS showing percent HEL^3X^-binding from tdTom^+^ SW_HEL_ GC B cells and PCs on d7 and d8 post-immunisation. Both populations are pre-gated on cells with sufficiently high BCR (Igk) levels for detecting HEL^3x^ binding. Full gating in Figure S1. C. Quantification of data in B. Every timepoint is pooled from 2-4 experiments, each with 2-5 mice. Each symbol represents a mouse. D. Experimental scheme for E-F E. Representative FACS showing percent HEL^3X^-binding of total and c-Myc^GFP+^ SW_HEL_ GC B cells (CD45.2^+^ IgD^low^ GL7^+^) on d7 and d8 post-immunisation. Cells are pre-gated as Igk^+^. F. Quantification of data in E. Every timepoint is pooled from 2 experiments, each with 2-5 mice. Each symbol pair represents one mouse. Two-tailed P values from unpaired t tests with Welch’s correction (C) and paired parametric t test (F): ****p<0.0001.

Naïve SW_HEL_ S1pr2^tdTom^ B cells (some with a Blimp1^mVenus^ PC reporter) were transferred into congenic WT hosts prior to HEL^3x^-sheep red blood cell (SRBC) immunisation to initiate GCs containing both transferred and endogenous B cells ^6,17^. Mice received tamoxifen on day 6 post-immunisation, with SW_HEL_ GCs and PCs analysed 1 and 2 days later (Figures 1A, B). Two independent PC gating approaches were used (Figures S1A-C) ^18^, giving consistent results. Consistent with Cre activity being GC-restricted, tdTom-labelled GC B cells but not PCs were evident within 24hrs (day 7), with small tdTom^+^ PCs populations appearing by day 8. This indicated a lag between commitment in the GC and the appearance of tdTom^+^ PCs, therefore the frequencies of HEL^3x+^ (high BCR affinity) cells among day 8 tdTom^+^ PCs and day 7 GCs were compared. This assay was possible because newly generated PCs retained surface immunoglobulin expression at this early post-GC stage (Figure S1A, B). TdTom^+^ PC populations were highly enriched for HEL^3x+^ cells (median 88%), whereas day 7 GCs mostly still expressed low affinity BCRs (7% HEL^3x+^), and remained far below PC antibody affinity equivalence at day 8 (45% HEL^3x+^) (Figures 1B, C, Figure S1D). The proportion that was HEL^3x+^ among GC B cells expressing a c-Myc-GFP reporter of positive selection ^19,20^, was also lower than in tdTom^+^ PCs, even when compared at the same time point (68% HEL^3x+^ for c-Myc-GFP^+^ at day 8, Figures 1D-F, Figure S1E). Therefore, these findings confirm that triggering thresholds differ for positive selection and PC differentiation ^21^, and support earlier conclusions that PC differentiation in SW_HEL_ GCs favours cells expressing higher affinity BCRs ^5,12^. However, an inherent limitation of the anti-HEL^3x^ response is that it matures extremely quickly; HEL^3x-^ cells that performed poorly at generating PCs on days 7-8 were also strongly outcompeted in GCs by day 9 (Figure 1C). This contrasts with polyclonal GC responses to complex antigens, where B cells expressing relatively low affinity BCRs persist for prolonged periods ^7,8,10^. Consequently, the question of how antigen receptor affinity impacts PC differentiation in such settings cannot be easily inferred and needs testing.

### Clonally inclusive plasma cell differentiation during influenza A infection

The GCs that form after challenges with pathogen-derived antigens involve many B cell clones that differ in their BCR affinities, binding footprints and epitope specificities ^7,8,10^. The situation is yet more complex during infection, where pathogens express multiple antigens and induce significant inflammation and tissue damage.

**Figure 2:**
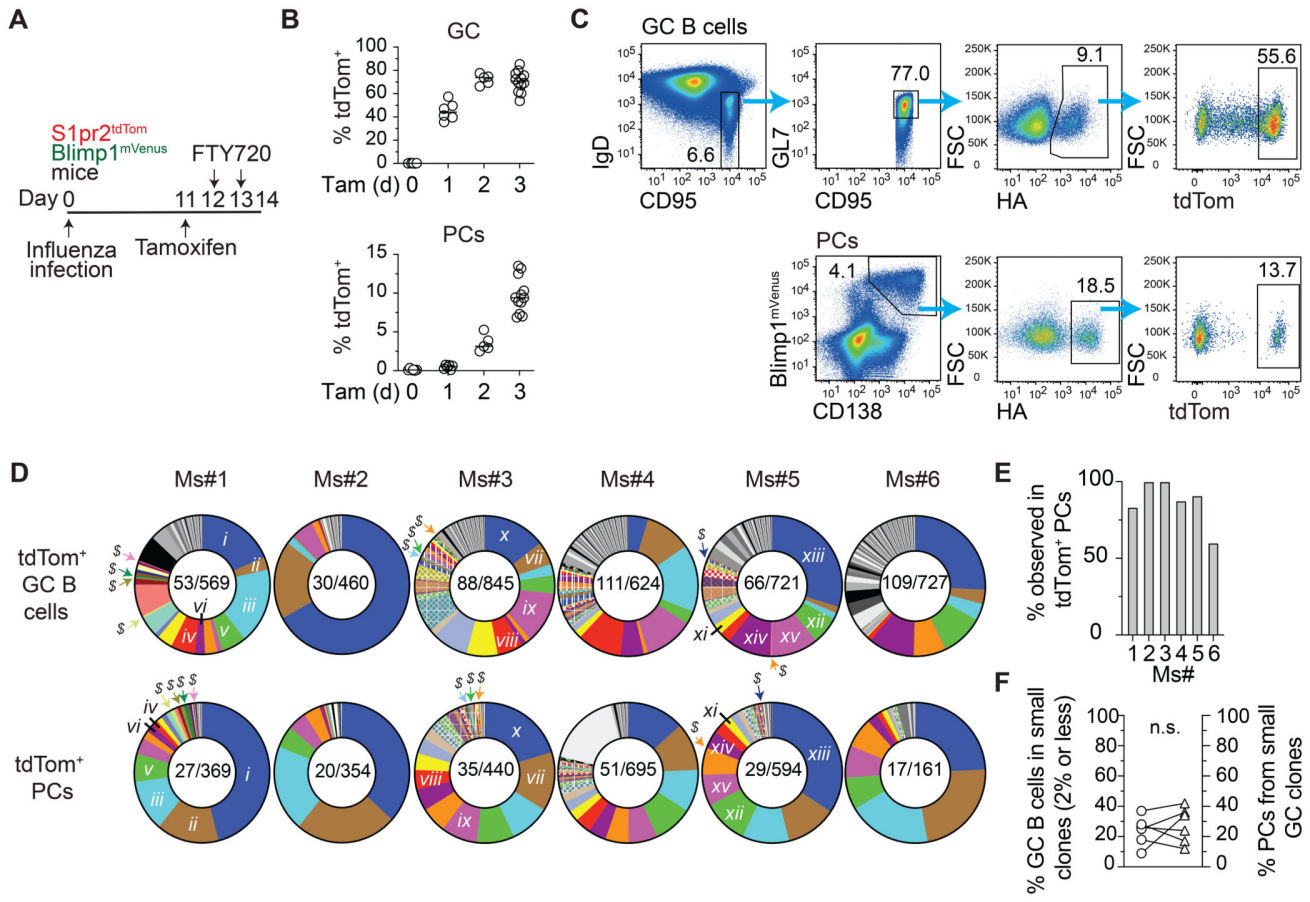
Clonality of plasma cell differentiation during influenza A infection A. Experimental scheme for B-F. B. Frequency of tdTom^+^ among day 14 MedLN GC B cells and PCs at the indicated time post-tamoxifen treatment. Each timepoint is pooled from 2 or more experiments, each with 2-4 mice. Gating strategy similar to C, except no HA gating and captures total (dim and bright) tdTom^+^ cells. C. Representative FACS gating for identifying (and sorting) HA^+^ tdTom^+^ GC B cells and PCs from MedLNs of S1pr2^tdTom^ Blimp1^mVenus^ mice. Day 14 post-infection shown. D. Pie charts showing the distribution into clonal lineages of tdTom^+^ GC B cells and PCs sorted as in C, for 6 mice. Each pie slice is a distinct clone. Coloured slices are lineages shared between the GC and PC compartments of a given mouse. Numbers: “Nb of clonal lineages detected/Nb of cells sequenced”. “$” signs indicate some subdominant clone examples found in tdTom^+^ PCs (only included for odd number mice to aid spacing). Roman numerals link clones to subsequent figures. E. Proportion of successful (representing >2% of total) GC clones detected in tdTom^+^ PCs. F. Proportion of GC B cells belonging to clonal lineages representing 2% or less of GC B cells, compared to the proportion of PCs from these clones. Each symbol pair represents one mouse. Two-tailed P value from paired parametric t test: n.s. p≥0.05.

To investigate PC differentiation in this setting, S1pr2^tdTom^ Blimp1^mVenus^ mice were infected with influenza A virus (HKx31, H3N2) and treated with a single tamoxifen injection 3 days prior to mediastinal lymph node (MedLN) harvest on day 14 (Figure 2A). FTY720 treatments were provided for the final 2 days to trap newly emerging PCs in the LNs where they develop ^22^. The kinetics of tdTom labelling again confirmed the lineage tracing fidelity, with PC labelling delayed by approximately one day (Figure 2B). As observed in SW_HEL_ experiments, tdTom^+^ PCs retained membrane immunoglobulins at this early stage (Figure S2A), therefore cells specific for haemagglutinin (HA) were identified using surface staining with a multimeric fluorescent probe, with comparisons made to contemporaneous GC B cells (Figure 2C).

HA-specific tdTom^+^ GC B cells and PCs from 6 mice were FACS sorted and their paired antibody heavy and light chain variable genes sequenced. Sorts were biased to capture the rarer tdTom^+^ PCs with greater depth relative to the more abundant GC B cells, thereby ensuring good PC coverage; 460-845 tdTom^+^ GC B cells and 161-695 tdTom^+^ PCs were analysed for each mouse. The distribution of antibody isotypes expressed by GC B cells and tdTom^+^ PCs was similar, although IgM and IgG3 expressing cells that were subdominant in GCs appeared to also generate slightly fewer PCs (Figures S2B), possibly reflecting a known relative defect in positive selection in the case of IgM-expressing GC cells ^23^. Unique clonal lineages were identified using heavy chain VDJ annotation and CDR3 sequence similarity (Figure 2D). As expected, cells in both subsets mostly carried multiple somatic mutations (Figure S2C), and most PCs (median 97%) could be traced to lineages also observed in GCs (coloured PC slices in Figure 2D). Overall, approximately one third of GC clonal lineages were detected within new PC populations (∼40 compared to ∼120, estimated using Chao1) ^8,24^, indicating an inevitable decrease in clonal diversity due to infrequent selection (Figure S2D). Despite this, almost all successful GC lineages (defined as representing >2% of GC B cells) were also found among tdTom^+^ PCs (coloured GC slices in Figure 2D, median of 89% across mice), arguing against the possibility that rare attributes or features are uniquely selected during PC differentiation (Figure 2E). Moreover, even lineages that performed poorly in GCs (defined as <1% GC B cells) were found in tdTom^+^ PC populations (some examples indicated by “*$*” signs for odd numbered mice, Figure 2D), and PC populations also contained sub-dominant lineages not detected in GCs (grey PC pie slices). Consequently, when the results were normalised for GC population size, successful and subdominant lineages (≤2% total GC) were similarly productive in generating PCs (Figure 2F), and no detectable trend existed for more successful GC clones seeding more PCs than is expected by abundance-based chance alone when observed results were compared to simulated random sampling (Figure S2E, F). Therefore, the overall probability of HA^+^ lineages generating PCs largely reflects their representation in GCs.

### Plasma cells emerge at all GC maturation stages as expanded “nodes”

**Figure 3:**
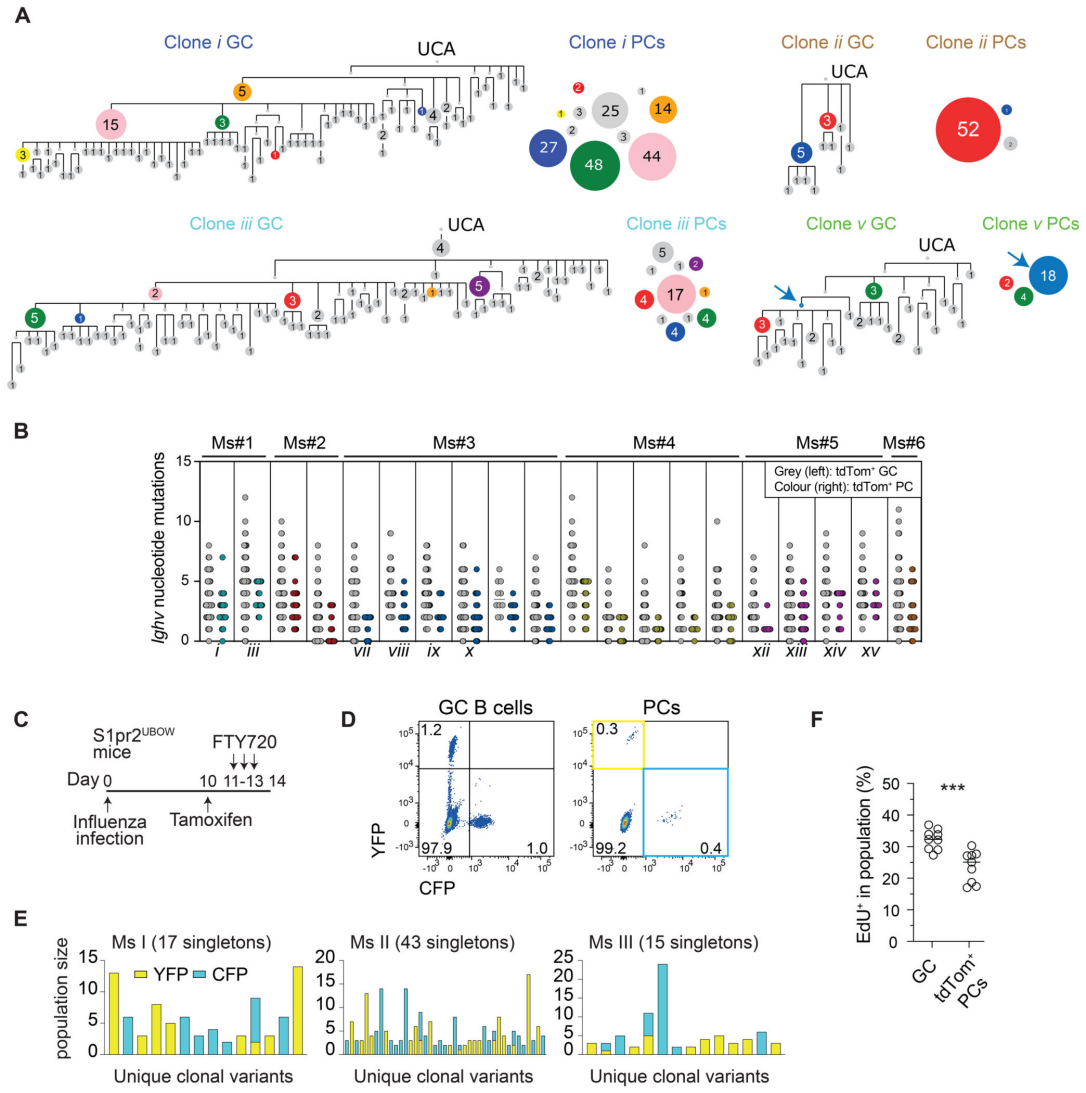
Plasma cells differentiate from multiple GC maturation stages and expand to generate “nodes” A. Tree representation of indicated clones from Ms#1 in Figure 2D, showing the phylogenetic relationship between GC B cells, and the observed population sizes for tdTom^+^ PCs. Coloured nodes indicate sequences observed in both GC and PC compartments. Numbers and node sizes indicate number of observed cells with identical HC VDJ sequences. Number-less nodes are inferred but not observed, arrows indicate where PC differentiation maps to them. Roman numerals relate to clonal lineages in Figure 2D. B. *Ighv* somatic mutation loads of GC B cells and PCs from clones where the PC compartment contains at least 5 unique VDJ sequences (across all 6 analysed mice). Each column is a clonal lineage. Roman numerals relate to clonal lineages in Figure 2D. C. Experimental scheme for D-E. D. Representative FACS of GC B cells (Dump/IgD^-^ B220^+^ CD95^+^ GL7^+^) and PCs (Dump/IgD^-^CD138^+^) in S1pr2^UBOW^ medLNs. E. PC node sizes (number of cells with identical HC VDJs), and the relative UBOW colours they derived from (bar colours). Sequences observed just once are not included as bars but their numbers are indicated (singletons). F. Experimental scheme as in Figure 2A but with mice receiving an EdU injection 1 hour before analysis. The percent of GC B cells and tdTom^+^ PCs that were EdU^+^ is shown. Two-tailed P value from paired parametric t test: ***p<0.001.

We interrogated PC differentiation within clonal lineages, with examples from Ms#1 (Figure 3A) and Ms#5 (Figure S3A) plotted (clones identifiable by roman numerals in Figure 2D). GC maturation trajectories were followed using inferred phylogenetic trees that reveal where somatic variants have branched (e.g., under unmutated common ancestor, UCA, in Ms#1 clone *i*, Figure 3A), or presumptively positively selected to expand and seed further diversification (“clonal bursts” ^8^, e.g., pink variant in same lineage) ^25^. Coloured somatic GC variants are sequences also found in tdTom^+^ PC populations, i.e., cells that differentiated, while grey cells were only observed in one subset. Notably, PCs developed from multiple levels within GC phylogeny, for example, Ms#1 clone *i* had PCs differentiating from 3 sequential GC expansions, including remnants of earlier bursts (orange and pink). Consequently, somatic mutation loads were similar or lower in tdTom^+^ PCs compared to GCs (Figure 3B). The findings that PCs emerged from multiple phylogenetic levels distanced by substantial expansions suggests that PC differentiation may not be restricted to only the very most matured GC cells, while concurrent differentiation from independent parallel lineage branches was also evident (e.g., Ms#1 clone *iii*, purple and pink). As such, GC B cells at different stages and branches of maturation pathways differentiate, generating PC populations that reflect the overall maturation process.

Differentiation gave rise to expanded populations, or “nodes”, of PCs with identical heavy chain VDJ genes and somatic mutations, sometimes numbering >50 cells in size (Figures 3A and S3A). Just over half of all PC sequences showed evidence of expansions (i.e., >1 PC with identical sequence), with a spread of node sizes (Figure S3B). This wide variation in node size may reflect the asynchronous nature of Cre activity as well as differential expansion. PC nodes commonly emerged from expanded maturation branch points in GCs (e.g., orange and pink for Ms#1 clone *i*), however other large nodes mapped to rarer GC clonal variants (e.g., blue in Ms#1 clone *i*), as well as to cells not detected in the GC (grey PC nodes). GC clonal “bursts” differ in their size and relative consequence for maturation pathways ^8,26^, therefore these results do not exclude a causative association between proliferative events and PC differentiation, however they do seemingly argue against an obligate link to strong “jackpot” selection events triggering extensive bursting and homogenisation. This conclusion is also supported by PCs emerging from GC expansions that are multiple phylogenetic levels up and so are unlikely to have been established in the period after tamoxifen treatment (e.g., orange for Ms#1 clone *i*).

PC nodes could in principle arise either by certain BCRs triggering multiple selection events or by a single PC-initiating selection event being linked to clonal expansion. To distinguish between these possibilities, similar *S1pr2*-CreERT2-based fate-mapping experiments were performed, but using LSL-Ubow mice in place of LSL-tdTom ^27^ (Figure 3C). Recombination is inefficient in this setting, labelling only ∼2% of GC B cells when assessed 4 days after tamoxifen treatment (Figure 3D). Despite this, sizable PC nodes of cells with identical heavy chain VDJ sequences were observed, most of which were of a single colour (61 of 68 nodes), indicating that single cells were selected for both clonal expansion and differentiation after Cre-mediated labelling (Figure 3E). This inferred post-selection proliferation might occur immediately before or after PC commitment, however tdTom^+^ PCs (in S1pr2^tdTom^ Blimp1^mVenus^ mice) were highly proliferative (∼25% 5-ethynyl-2’-deoxyuridine (EdU)^+^ in 1hr, Figure 3F), confirming an important role for the latter. Therefore, GCs output nodes of PCs expressing identical antibodies through associated processes of differentiation and expansion.

### GCs support plasma cell differentiation by low and high affinity B cells

**Figure 4:**
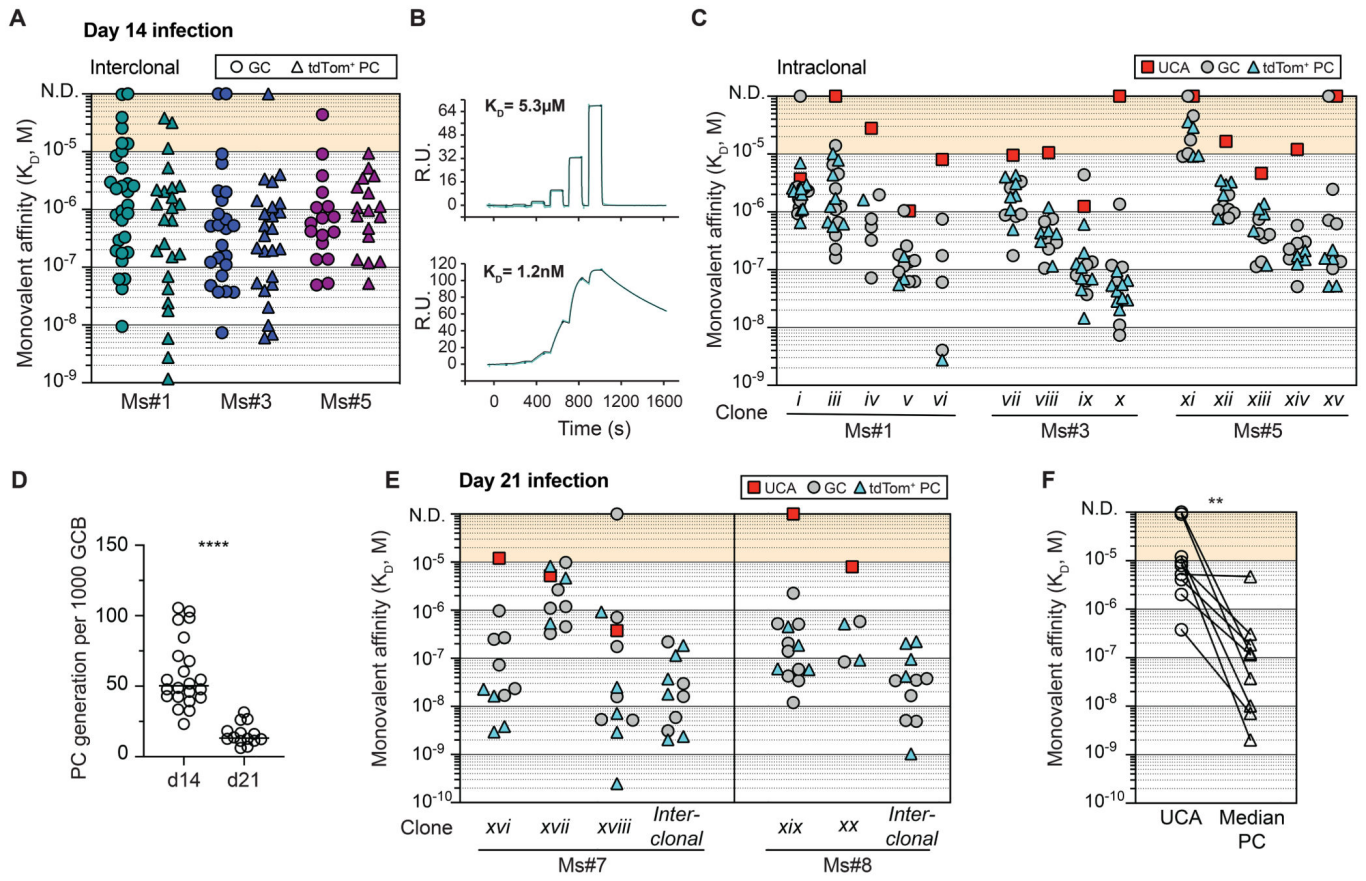
GC B cells expressing low and high affinity BCRs develop into plasma cells A, C, E and F. Affinities (K_Ds_) for monovalent Fab binding to recombinant HA. Each symbol represents a Fab derived from a single B cell. An accuracy threshold of 10µM is indicated (shading); detectable binding occurred above this but measurements are less accurate (see methods). N.D. = binding but no K_D_ determined. Some N.D. Fabs bound antigen when reconstituted as surface IgMs but not by SPR. A. Fab K_D_s from GC B cells and PCs picked from various clones for Ms#1, Ms#3 and Ms#5 from Figure 2. Cells were picked as to sample across a broad range of clonal lineages, rather than be just random, to avoid excessively biasing for abundant lineages. B. Example SPR single-cycle kinetic traces from PC Fabs from Ms#1. C. Fab K_D_s for GC B cells and PCs from the indicated expanded clonal lineages. Roman numerals relate to clonal lineages of Figure 2D. PCs were selected from larger PC nodes, while GC were picked as a sampling across “grey” nodes, i.e., not detected among PCs. Note that most PC sequences were also found in GC (but are not shown as GC K_D_s). Some K_D_s from clone *v* were measured using biolayer interferometry. D. PC generation efficiency (number of tdTom^+^ PCs per 1000 tdTom^+^ GC B cells) in medLNs harvested on d14 or d21 post-infection. Each timepoint is a pool of at least 3 experiments, each with 2-5 mice. Each symbol is one mouse. E. Fab K_D_s for GC B cells and PCs sorted on d21 post-infection, following a 3d-tamoxifen treatment. Roman numerals relate to clonal lineages of figure S4. Interclonal cells are picked from across other clonal lineages. F. Comparison of the median K_D_s of d21 post-infection PCs from various clonal lineages to the K_D_s of inferred UCAs. Each pair of symbols represents a clonal lineage. Two-tailed P values from Mann Whitney test (D) and Wilcoxon matched-pairs signed rank test (F): **p<0.01, ****p<0.0001.

To investigate how antibody affinity impacts GC PC differentiation during infection, paired heavy and light chain variable region genes from day 14 influenza infection HA-specific tdTom^+^ GC B cells and PCs were cloned and expressed as Fabs, for three independent mice. Monovalent binding affinities were determined using surface plasmon resonance (SPR), with recombinant HA trimer immobilised and Fab analytes. An initial analysis focussed on Fabs derived from cells picked across lineages to acquire an overview of the spread of antibody affinities present within the confines of paired sequence recovery. Consistent with previous findings ^8,10^, individual GC B cells from different lineages differed substantially in their antibody affinities (multiple 1000s-fold), with most spanning ∼100-200-fold range (Figure 4A, B). Surprisingly, the affinity spread observed among tdTom^+^ PCs was similar to that of GCs. Both populations contained cells with “good” nanomolar-range antibody affinities but also cells whose Fabs bound the antigen (either by SPR or by staining reconstituted surface IgMs ^28^) with affinities that were too low to accurately measure (K_D_ > 10µM). Importantly, this means that GC B cells with weak affinity BCRs differentiated even when competing with cells expressing much higher affinity antigen receptors.

We next explored the relationship between BCR affinity and PC differentiation within clonal lineages, studying 4-5 unique clones for each mouse (Figure 4C). Individual clones supported affinity ranges that were considerable (∼10-30 fold) but less broad than between lineages, as expected ^10^. A comparison of somatic variants to UCAs supports that GCs read and promote relatively modest affinity enhancements through positive selection (e.g., Ms#1 clone *i*, Ms#3 clone *vii* and Ms#5 clone *xiii*). Despite this, tdTom^+^ PC Fabs approximately mirrored GC B cells from the same lineages in terms of their affinities, including for clones containing relatively broad ranges (e.g., Ms#3, clone *ix* with K_D_s spanning ∼40-fold), and lower affinity clones (e.g., Ms#1 clone *iii*, Ms#5 clone *xi*). This explains why PCs emerged from early and late maturation stages in GC trees (Figures 3A and S3A). The intraclonal analysis also adds important granularity to the interclonal picture painted (Fig. 4A), by showing that PCs simultaneously differentiate from co-maturing lineages despite the differing absolute affinities of their antibodies. This explains why PC differentiation was broadly permissive across clonal lineages, including those that were subdominant in GCs (Figure 2D). In fact, some of the most PC productive GC lineages displayed moderate antibody binding affinities relative to coexisting clones (e.g., clones *i* vs *v* in Ms#1, and clones *vii* vs *ix* in Ms#3).

We considered whether our findings might be unique to relatively early-stage anti-viral responses, by performing a similar analysis on day 21. Overall PC output, normalised to GC size, was reduced by ∼4 fold (Figure 4D), which led to slightly lower tdTom^+^ PC diversity in terms both of number of distinct lineages and somatic variants (Figure S4A, average 17 PC lineages observed compared to 30 at day 14). However, similar selection principles were evident, with PCs again emerging as nodes derived from both immunodominant and subdominant GC clones, although one mouse showed evidence of a strong clonal bias (Ms#9) (Figures S4A-C). The overall response affinity was much improved with the additional week of maturation (∼20nM compared to ∼600nM range), however large interclonal affinity spreads were still evident in the GC and conserved during PC differentiation (e.g., clones *xvi* and *xvii* in Ms #7, both generating PCs despite clone *xvii* being subdominant, Figure 4E, S4A). Within clonal lineages, PCs that matched the lowest GC antibody affinities were still found, and differentiation again occurred from multiple levels of the maturation pathway (Figure S4B). Some clones uniformly carried fewer mutations (e.g., Ms#7 clone *xvii*, which was also of comparatively low affinity), possibly suggesting that cells infiltrating established GCs had successfully begun making PCs (Figure S4D) ^29,30^. Therefore, late-stage GCs generated fewer PCs but with similar selection rules. Importantly, even PCs from relatively weak antibody affinity clones were much improved compared to their UCAs (Figure 4F), indicating an evolutionary advantage to selecting PCs this way.

### Tracking plasma cell differentiation to single GCs

**Figure 5:**
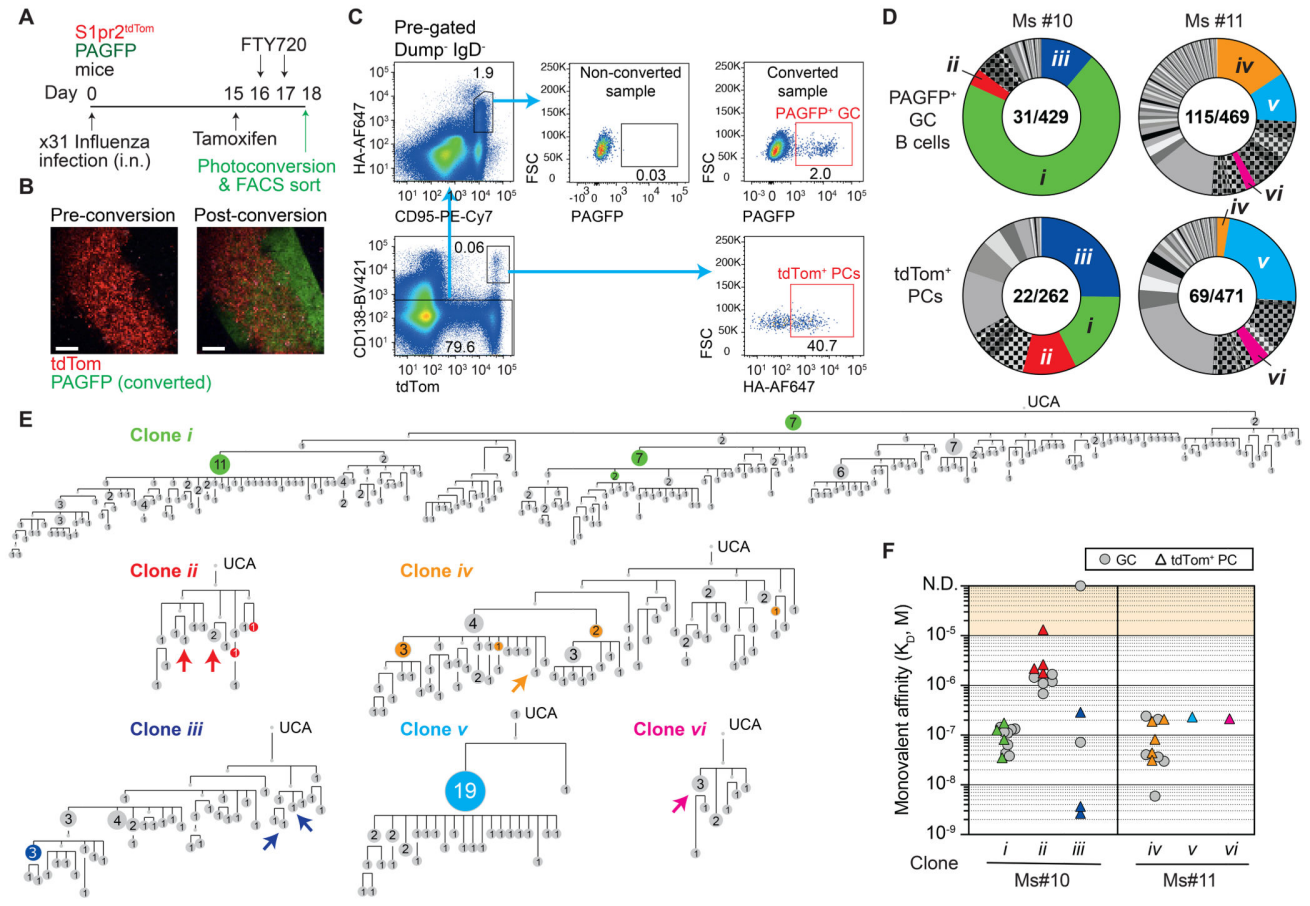
Tracking plasma cell differentiation to single GCs A. Experimental scheme for B-F. B. Multi-photon microscopy image of a single tdTom^+^ GC pre- and post-photoconversion. Scale bar, 100µm. C. Representative FACS gates for sorting HA^+^ tdTom^+^ PCs and HA^+^ PAGFP^+^ single GC B cells. D. Pie charts showing the distribution of clonal lineages from two mice where PCs could be traced to individual GCs. Coloured slices indicate clones found among GC and PC compartments, and in which individual PCs were traced to the GC on the basis of shared SHM patterns. Hashed slices indicate lineages shared between the GC and PC compartments, but where no PCs could be confidently traced to the converted GC. Numbers indicate: “Nb of clonal lineages/Nb of cells sequenced”. E. Phylogenetic maturation trees (GC) for clones labelled in D. Coloured nodes indicate where tdTom^+^ PCs with identical HC VDJ sequences were observed. Arrows indicate the nearest cell in the tree to tdTom^+^ PCs sharing 3 or more HC VDJ somatic mutations with PAGFP^+^ GC B cells. F. Monovalent affinities (K_D_s) for HA of Fabs from indicated clones (GC and PCs). Each symbol is a single B cell Fab. An accuracy threshold of 10µM is indicated by shading; binding was detected above this but measurements considered less accurate (see methods). N.D. = binding but no K_D_ determined. PCs were picked as in E, GC were picked from across “grey” nodes.

Affinity maturing B cells do not typically move between GCs, therefore their evolution and fate commitment occurs in partially isolated islands that may differ in selection criteria ^8,31^. We therefore explored whether concurrent low and high affinity PC generation holds true in single GCs. We adopted a 2-photon microscopy-mediated photoactivatable GFP (PAGFP) approach ^8,32^ to mark individual GCs *in situ* and subsequently FACS sort them (Figure 5A-C). S1pr2^tdTom^ labelling facilitated GC identification for conversion and again marked new PCs. PCs were not photoconverted, however their origin in PAGFP^+^ GCs was assigned based on clonality and shared somatic mutation patterns, i.e., where identical mutated sequences were observed in both populations, or where multiple somatic mutations were shared.

PCs were successfully traced to 2 GCs, with coloured GC pie slices (Figure 5D), and coloured GC tree variants (Figure 5E), marking where differentiation occurred. PCs differentiated both from dominant winner clones (e.g., Ms#10 Clone *i*), and from those competing less well in the GC (e.g., Ms#10 Clone *ii*, Ms#11 Clones *vi*). Fabs were expressed for multiple GC B cells and PCs from Ms#10 clones *i-iii*, as well as Ms#11 clones *iv-vi*, and their affinities compared (Figure 5F). Clones *i* and *ii* from Ms#10 had median affinity differences of ∼15-fold (116 nM vs 1.8 µM), yet both generated PCs. The highest and lowest antibody affinity PCs traced to this single GC were 2.7 nM and 13 µM, suggesting that it concurrently generated PCs with antibody affinity differences of ∼4000-fold. Although the affinity spread observed among the 3 clones studied for Ms#11 was more modest, tdTom^+^ PCs ∼35-fold lower in antibody affinity than the best GC B cells were still observed (equivalent to the full range measured). As such, individual GCs seemingly support side-by-side maturation and PC differentiation of B cells with substantially different antibody affinities.

### Immunisation-induced GCs are less productive but still support the development of plasma cells with disparate antibody affinities

**Figure 6:**
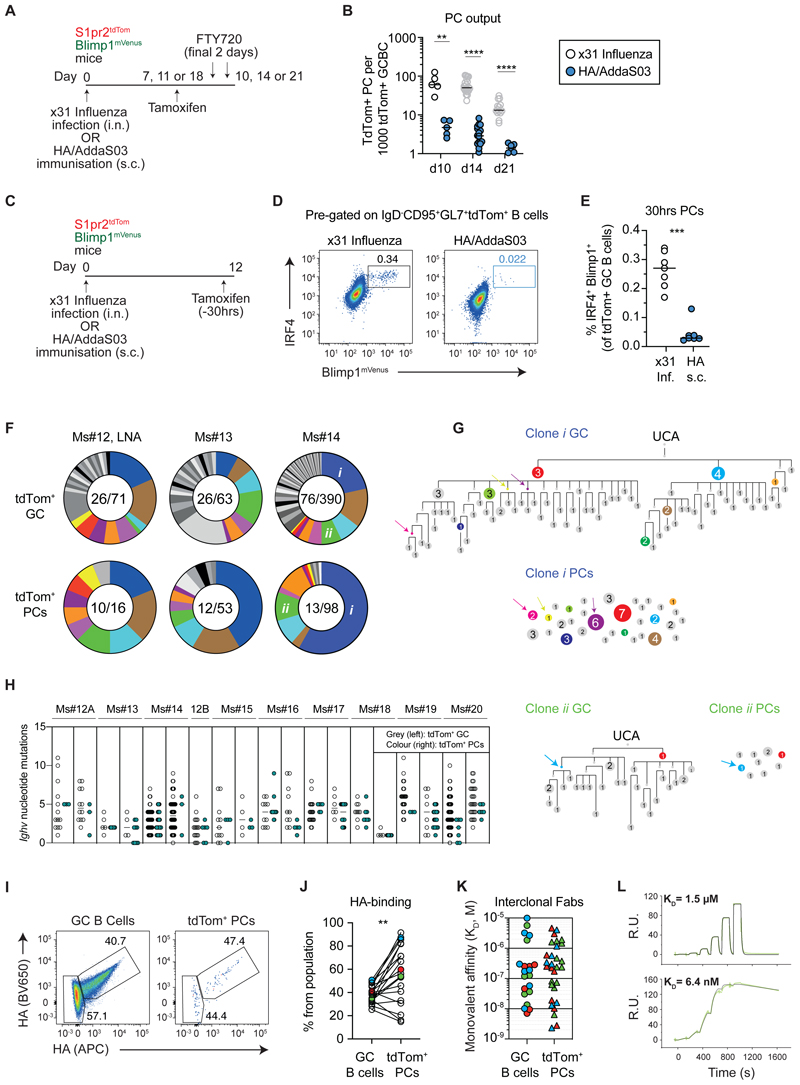
GCs output fewer plasma cells after immunisation but these still display disparate antibody affinities A. Experimental scheme for B. B. GC size-normalised PC output (number of tdTom^+^ PCs per 1000 tdTom^+^ GC B cells) at the indicated timepoints following infection or immunisation. Each symbol is a mouse. Each condition is pooled from at least 2 experiments, each with 2-5 mice, except d10 results that are each from one experiment with 5 mice. The grey data points are the same as in Figure 4D but are included for comparison. C. Experimental scheme for D-E. D. Representative FACS showing the proportion of Blimp1^+^ IRF4^+^ cells among tdTom^+^ GC B cells. E. Quantification of D. Each symbol is a mouse. Data pooled from 2 experiments, each with 3-4 mice per condition. F. Antibody genes from tdTom^+^ GC B cells and PCs were sequenced on d14 post-HA/AddaS03 immunisation. Pies show the distribution into clonal lineages, each slice is a distinct clone. Coloured lineages are shared between tdTom^+^ GC and PC compartments of a given mouse. Numbers: “Nb of clonal lineages detected/Nb of cells sequenced”. Similar analysis for a further 7 LNs is included in Figure S5E. G. Phylogenetic maturation trees for clones labelled in F and observed population sizes for tdTom^+^ PCs. Coloured nodes indicate sequences observed in both GC and PCs. Numbers and node sizes indicate number of observed cells with identical HC VDJ sequences. Number-less nodes are inferred but not observed, arrows indicate where PC differentiation maps to them. H. *Ighv* somatic mutation loads of GC B cells and tdTom^+^ PCs from the two most immunodominant clones in the GC across all 10 analysed LNs (Ms#12B-20 in Figure S5). Each column is a clonal lineage. I. Representative FACS showing the proportion of HA-binding and non-binding cells among GC B cells and tdTom^+^ PCs. Gates as in Fig. 2B, includes pre-gating on Igk^+^. Probes are tetramerized trimers. J. Quantification of I. Each symbol pair is one mouse (means reported where iLNs analysed individually). Data pooled from 6 experiments, each with 2-4 mice. Results include experiments using a trimeric HA probes and tetramerised trimeric HA probes, and not all include Igk^+^ gating. K. K_Ds_ for HA binding by Fabs from the indicated populations, reflecting various clones. Each symbol represents a single B cell Fab, colours indicate different mice and are linked to J. L. SPR single-cycle kinetic traces from high and low affinity PC Fabs, from one mouse in K. Two-tailed P values from Mann Whitney tests (B, E), and paired parametric t test (J): **p<0.01, ***p<0.001, ****p<0.0001.

We explored whether similar PC selection rules apply in GCs formed in response to subunit vaccination. S1pr2^tdTom^ Blimp1^mVenus^ mice were immunised subcutaneously (s.c.) with recombinant HA protein in an AS03-like adjuvant (AddaS03), and the newly emerging PCs were again characterised (Figure 6A). PC production efficiency after immunisation was strikingly reduced, with GCs generating far fewer PCs than during infection, even when results were normalised for GC size (17-fold fewer on day 14, Figure 6B). Similar findings were made using an independent genetic fate-mapping model (*Aicda*-CreERT2 Rosa26-LSL-tdTom mice, Figure S5A) ^33^, at different time points (Figure 6B), and after immunisation with a different subunit vaccine (Chicken Gamma Globulin/Alum, Figure S5B). We examined whether the lower PC output after immunisation might reflect an absence of post-GC PC proliferation, however the frequency of tdTom^+^ PCs incorporating EdU was similar to during infection (median 29%, Figure S5C). The possibility that PC differentiation occurs more frequently in infection GCs was therefore considered. Additional GC fate-mapping experiments but with shorter (30hr) tamoxifen treatments were performed, following the rationale that this should allow some differentiation but minimise secondary effects from proliferation and/or apoptosis (Figure 6C). The proportion of tdTom^+^ GC-phenotype cells that were IRF4^high^ and Blimp1^+^ was much higher during infection, despite similar tdTom-labelling kinetics (Figures 6D, E, S5D), consistent with differentiation being initiated more frequently in that setting.

Despite immunisation-induced GCs developing fewer PCs, subdominant as well as dominant GC clonal lineages again underwent differentiation (Figure 6F, subdominant examples include Ms #12 LNA green clone, Ms #13 purple clone, Ms#14 red, yellow and pink clones, grey PC clones in all mice). A small number of clones outputted more PC nodes than expected from abundance alone (Figure S5F), possibly reflecting periods of preferential differentiation. PC differentiation again approximately mirrored the overall clonal maturation process, because tdTom^+^ PC sequences mapped to multiple levels in phylogeny, and *Ighv* somatic mutation spreads were similar for the two subsets (Figures 6G, H, S5G). PC nodes generated from immunisation GCs were notably smaller than those from infection (Figure S5H), despite their similar EdU incorporation rates (Figure S5C), likely indicating reduced post-GC survival, although expression of a lineage specific anti-apoptotic Bcl2 transgene did not detectably increase output efficiency (Figures S5I, J) ^34^. Ultimately, the reduced PC productivity of GCs after immunisation will result in them contributing less serum antibody.

Several groups have reported that GCs formed after immunisation with adjuvanted pure protein contain sizeable populations (often ∼50%) of cells that do not detectably bind the immunising multimeric antigen by FACS or ELISA/BLI assays, with these thought to have antibody affinities below detection limits and/or to target partially degraded or modified antigen forms (“dark antigen”) ^7,10^. HA FACS probe negative (HA^-^) cells were evident in GCs from HA/AddaS03 immunised mice, and they also became tdTom^+^ PCs (Figure 6I, J). The relative efficiency with which HA^-^ cells differentiated varied between mice, however HA^+^ GC cells generated significantly more PCs when all mice/experiments were pooled (median 39% HA^+^ in GC, compared to 56% in tdTom^+^ PCs, Figure 6J). The presence in GC populations of cells carrying damaging or dead-end mutations may contribute to this effect ^35,36^, however it probably also reflects preferential (but not exclusive) differentiation of cells that reach minimal affinity thresholds to confer detectable binding of HA multimers.

Finally, we measured the affinities of HA-binding Fabs from GC B cells and tdTom^+^ PCs for 3 mice. This analysis was less comprehensive than the one performed after infection, however it allowed us to explore the ranges of affinities supported through PC differentiation. When Fabs from across clonal lineages were analysed, the antibody affinities of GC B cells and tdTom^+^ PCs both spanned multiple orders of magnitude (>1000-fold) (Figures 6K, L). As such, PC differentiation after immunisation is inclusive of broad affinity ranges, including cells that were unable to bind multimeric HA probes. Collectively, our results from infection and immunisation indicate that the selective criteria driving GC PC differentiation approximately tracks that driving affinity maturation.

## Discussion

Our study reveals how antibody affinity and GC B cell clonality impact GC PC differentiation during complex responses. Studies of the clonally-restricted anti-HEL^3x^ response had indicated a strong bias towards only high affinity GC B cells differentiating to become PCs (^5^, as well as Fig. 1). In contrast, we report that the GCs formed after influenza infection co-mature B cell clones of vastly different antibody affinity alongside each other and output PC populations that largely mirror the contemporaneous (or very soon-to-be) GC. Surprisingly, this means that GCs generate PCs expressing antibodies that might be considered of weak affinity, explaining recent findings from West Nile Virus immune mice ^37^. We therefore conclude that PC selection is not unduly restrictive and instead broadly reflects the selection environment driving the affinity maturation process. Our experiments also revealed that, although PC differentiation occurs infrequently in GCs, it is commonly associated with extensive clonal expansions that involve PC proliferation. These will enhance antibody secretion and may also improve the chances of PCs reaching distant supportive niches for long-term survival. Our experimental regimens all included FTY720 treatments, however it is plausible that clonal expansion could continue after LN egress without S1PR1 antagonism.

The idea that only GC B cells expressing the highest affinity BCRs are selected for PC differentiation was appealing because their antibodies are expected to functionally saturate their targets at lower concentrations, all else being equal. However, antibody potency, even among neutralisers, varies greatly depending upon the epitopes bound and the molecular interactions involved. For these reasons, correlations between antibody affinity and potency can be quite poor when considered across clones, as demonstrated for SARS-CoV2 ^38^. B cells have no way of measuring epitope potency and so we propose that it makes evolutionary sense for GCs to co-mature clones of different affinities while shuttling broad samplings into PC pools without excess bias. Moreover, due to their bivalent nature, antibodies that have reasonably good association rates and bind with both arms may in practice gain little advantage by improving beyond moderate affinities (e.g., K_Ds_ ∼100-10nM and better) when their targets are only transiently exposed, such as virions moving between cells ^39,40^. This selection approach would also help ensure redundancy in epitopes and binding modes, countering pathogen immune escape. Notably, even relatively weak Fabs on day 21 were much improved compared to their predicted UCAs, meaning that there was a global improvement in the response.

Our findings from infection and subunit immunisation could be interpreted as arguing against models where PC differentiation depends on selection signal strength, however we instead believe that they probably reflect the biological outcome of “noisy” affinity-based selection in physiological settings. SW_HEL_ experiments mimic the intraclonal setting but have the notable feature that the stereotypical Y53 mutation confers an affinity leap (∼100-fold) and is associated with near unifying GC selection very early after immunisation (within ∼10 days). This may amplify an effect that is somewhat muted in more mature GCs, where affinity enhancements tend to be gradual and selection less accurate, which may reduce competition-dependent repression. Potential sources of “noise” include the unequal composition of immune complexes ^41^, stochastic or inherited differences in B cell signalling or transcriptional states, the presence of different T cell specificities or activation statuses ^42^, cross-talk from innate sensing mechanisms (e.g., TLRs or complement receptors ^43,44^), and variations in LZ dwell time ^45^. Our findings confirmed that the triggering threshold for PC differentiation differs from that inducing expression of the selection marker c-Myc, however this may be necessary for achieving similar outcomes, because positive selection occurs iteratively. This argument is supported by the finding that SW_HEL_ tdTom^+^ PCs are strongly enriched for high affinity cells, but that GCs almost catch up within two days.

The above arguments alone probably do not explain how PCs develop from different clonal lineages whose K_D_s differ by multiple orders of magnitude. We explored the possibility that this reflects partitioning of responses into low and high affinity GC islands, however the experimental evidence did not support this as the sole cause. Rather, we speculate that complexities of *in vivo* antigen sensing may mean that B cells simply do not discern these large interclonal affinity differences ^46^. For example, epitope accessibility may differ due to steric hindrance or antibody feedback ^47–49^, antigen presentation efficiency might vary among clones ^42^, and binding kinetics could differ when immobilised antigen complexes are sensed by membrane BCRs under tensional forces ^50–52^. Ultimately, if these factors allow GCs to apply affinity discrimination more tightly within than between lineages, they will represent elegant evolutionary solutions for supporting antibody responses that affinity-mature while remaining diverse.

The mechanism responsible for triggering PC commitment in GCs remains to be worked out. The striking association with BCR affinity in clonally restricted settings has led to suggestions that PC differentiation occurs as an obligate alternative fate choice to continued participation in GCs when LZ cells receive very strong selection inputs. One conceptual concern with models involving bifurcation at this stage is the risk of GCs depleting themselves of high BCR affinity cells immediately after their generation ^3^. Experimental evidence also supports that strong selection inputs concurrently promote both fates rather than one at the expense of the other ^32^. An alternate possibility therefore is that PCs (or cells sensitised for this fate) might develop as a secondary product of the proliferative events that shape affinity maturation ^3,41^. In this scenario, daughter cells could branch away from GC expansions and be predisposed towards the PC fate. This could explain why Blimp1 induction in a fraction of GC B cells is possible following BCR engagement without T cell help ^12^, despite the presumed scarcity of antigen on follicular dendritic cells and the muted BCR signalling capacity of GC B cells ^53–55^. Recent evidence that IL-21 “re-wires” selection towards promoting PC differentiation ^13,56^, and that this T cell-derived cytokine acts outside of immune synapses ^57^, might provide a bridge between stochastic and instructive processes, because it implies that a cell’s fate may also be tied to its local microenvironment.

Our findings have implications for rational vaccine design. There has been renewed optimism in recent years about the possibility of generating protective vaccines for certain challenging pathogens through reverse vaccinology 2.0. ^58^, an approach involving the engineering of immunogens to coax maturation pathways that ultimately mould antibodies similar to rare protective versions from infected patients (e.g., HIV broadly neutralising antibodies). One concern is that successfully matured cells may need to compete strongly against all non-protective lineages for them to seed serum antibodies, however our findings are reassuring in that they suggest that most clones contributing significantly to GCs should also generate PCs – albeit in approximate accord with their relative immunodominance. Our results also highlight that the number of PCs made in GCs can vary dramatically with the immune context. These productivity differences are not easily explained by our current understanding of selection events and therefore additional studies are needed to decipher their underlying mechanisms. Whether different vaccine platforms, such as mRNA- or viral vector-based approaches, will behave closer to infection or protein immunisation in terms of PC productivity also merits further investigation.

### Limitations of the study

Our study sampled cells (for Fab characterisation) within and between clones on a moderately large scale, however it was not possible to do this for every cell. As such, the results provide an overview of affinities present in GC and new PC populations, but should not be read as a fully quantitative comparison of the average antibody affinities. We conclude that the spreads of antibody affinities encoded by HA^+^ GC and tdTom^+^ PC populations were similar, not that they are identical. The time lag inherent to fate-mapping also limits the comparisons, as well as the resolution with which PC differentiation can be assigned to GC phylogeny.

We conclude that the antibody affinity threshold for induction of the c-Myc marker of positive selection differs to that for PC differentiation, based on comparative SW_HEL_ experiments. We did not assess other selection-associated pathways such as mTorc1 activation or CylinD3 induction ^59,60^. Various selection pathways operate in concert in non-binary dose-dependent manners to drive affinity maturation via sequential clonal bursts ^45,61,62^, and so while c-Myc induction is a marker of positive selection ^19,20^, its expression alone does not provide a measure of the selective pressure driving affinity maturation. These clonally-restricted responses affinity-mature very quickly and so we also cannot exclude that selection rules differ at different response stages.

## Star Methods

### KEY RESOURCES TABLE

**Table:** 

REAGENT or RESOURCE	SOURCE	IDENTIFIER
Antibodies		
BV785-B220, clone RA3-6B2	Biolegend	Cat#103246, RRID:AB_2563256
BV605-B220, clone RA3-6B2	Biolegend	Cat#103244, RRID:AB_2563312
PE-B220, clone RA3-6B2	Biolegend	Cat#103208, RRID:AB_312993
Biotin - IgD, clone 11-26c.2a	Biolegend	Cat#405734, RRID:AB_2563344
BV605-IgD, clone 11-26c.2a	Biolegend	Cat#405727, RRID:AB_2562887
BUV395-IgD, clone 11-26c.2a	BD Biosciences	Cat#565988, RRID:AB_2737433
PECy7-CD95, clone Jo2	BD Biosciences	Cat#557653, RRID:AB_396768
Pacific Blue-GL7, clone GL7	Biolegend	Cat#144614, RRID:AB_2563292
FITC-GL7, clone GL7	Biolegend	Cat#144604, RRID:AB_2561697
AF647-GL7, clone GL7	Biolegend	Cat#144606, RRID:AB_2562185
Biotin-GL7, clone GL7	eBioscience	Cat#13-5902-82, RRID:AB_823151
PerCPCy5.5-GL7, clone GL7	Biolegend	Cat#144610, RRID:AB_2562979
BV421-CD138, clone 281-2	Biolegend	Cat#142507, RRID:AB_11204257
PE-CD138, clone 281-2	Biolegend	Cat#142504, RRID:AB_10916119
APCCy7-CD4, clone GK1.5	Biolegend	Cat#100413, RRID:AB_312698
APCCy7-CD8a, clone 53-6.7	Biolegend	Cat#100713, RRID:AB_312752
APCCy7-TER119, clone TER-119	Biolegend	Cat#116223, RRID:AB_2137788
APCCy7-F4/80, clone BM8	Biolegend	Cat#123117, RRID:AB_893489
Biotin-CD267, clone 8F10	Miltenyi Biotech	Cat#130-101-980, RRID:AB_2656779
AF647-IRF4, clone IRF4.3E4	Biolegend	Cat#646408, RRID:AB_2564048
Purified CD16/32, clone 93	Biolegend	Cat#101302, RRID:AB_312801
BUV395-IgKappa, clone 187.1	BD Biosciences	Cat#742839, RRID:AB_2741090
APCCy7-IgKappa, clone RMK-45	Biolegend	Cat#409504, RRID:AB_2563579
BV785-CD45.1, clone A20	Biolegend	Cat#110743, RRID:AB_2563379
BUV395-CD45.2, clone 104	BD Biosciences	Cat#564616, RRID:AB_2738867
Bacterial and virus strains		
HKx31 H3N2 Influenza A virus (grown in MDCK cells)	This paper	N/A
Chemicals, peptides and recombinant proteins		
AddaS03	Invivogen	Cat#vac-as03-10
Alhydrogel	Invivogen	Cat#vac-alu-250
Sigma Adjuvant system	Sigma Aldrich	Cat#S6322-1VL
Lipopolysaccharide	Sigma Aldrich	Cat#L6529
Tamoxifen	Sigma Aldrich	Cat#T5648-1G
Fingolimod (FTY720) HCl	Selleckchem	Cat#S5002
EdU	Biosynth Carbosynth	Cat#NE08701
2-Mercaptoethanol	Merck	Cat#63689-25ML-F
D-Mannitol	Sigma Aldrich	Cat#M4125-500G
Fixable viability dye efluor780	Biolegend	Cat#65-0865-14
Stretavidin-Qdot605	Thermo Fisher Scientific	Cat#Q10101MP
Streptavidin-BV605	Biolegend	Cat#405229
Streptavidin-BV650	Biolegend	Cat#405231
Streptavidin-APC	Biolegend	Cat#405207
Chicken Gamma Globulin	Rockland	Cat#D602-0100
HKx31 H3N2 Influenza A virus WT	This paper	N/A
HKx31 H3N2 Influenza A virus Y98F	This paper	N/A
Lysozyme from chicken egg white (Hen Egg Lysozyme, HEL)	Sigma Aldrich	Cat#L6876-5g
Critical commercial assays		
AF647, Alexa Fluor 647 protein labelling kit	Thermo Fisher Scientific	Cat#A20186
Biotin-X-NHS	Clabiochem	Cat#203188-25MG
FoxP3/Transcription Factor Staining buffer kit	Thermo Fisher Scientific	Cat#00-8333-56
BD fixation/permeabilization solution kit	BD Biosciences	Cat#51-2090KZ
Click-iT™ Plus EdU Alexa Fluor™ 647 Flow Cytometry Assay Kit	Thermo Fisher Scientific	Cat#C10634
Biotin CAPture kit, Series S	Cytiva	Cat#28920234
Miseq Reagent Nano Kit v2 (500 cycles)	Illumina	Cat#MS-103-1003
NextSeq 500/550 Mid Output Kit v2.5 (150 cycles)	Illumina	Cat#20024904
Qubit HS DNA assay	Thermo Fisher Scientific	Cat#Q32851
Bioanalyzer High Sensitivity DNA Kit	Agilent Technologies	Cat#5067-4626
Chromium Next GEM Single Cell 5’ Kit v2, 4 rxns	10X Genomics	Cat#1000265
Chromium Next GEM Chip K Single Cell Kit, 16 rxns	10X Genomics	Cat#1000287
Chromium Single Cell Mouse BCR Amplification Kit, 16 rxns	10X Genomics	Cat#1000255
Dual Index Kit TT Set A 96 rxns	10X Genomics	Cat#1000215
5’ Feature Barcode Kit, 16 rxns	10X Genomics	Cat#1000256
Dual Index Kit TN Set A, 96 rxn	10X Genomics	Cat#1000250
Library Construction Kit, 16 rxns	10X Genomics	Cat#1000190
ExpiFectamine 293 Transfection Kit	Gibco	Cat#A14524
Experimental models: Cell lines		
Expi293 cells	Thermo Fisher	Cat#A14527
ExpiCHO cells	Thermo Fisher	Cat#A29127
Experimental models: Organisms/strains		
Mouse: C57BL/6	Oxford University core breeding facility	N/A
Mouse: B6.SJL-*Ptprc*^*a*^ *Pepc*^*b*^/BoyJ	Oxford University core breeding facility	N/A
Mouse: SW_HEL_	Phan et al., 2003	N/A
Mouse: Rosa26-LSL-tdTom: B6.Cg-*Gt(ROSA)26Sor*^*tm9(CAG-tdTomato)Hze*^/J	Madisen et al., 2010	Jax. 007990
Mouse: S1pr2-CreERT2	Shinnakasu et al., 2016	N/A
Mouse: Blimp1-mVenus	Ohinata et al., 2018	N/A
Mouse: UBOW	Ghigo et al., 2013	N/A
Mouse: PAGFP: B6.Cg-*Ptprc*^*a*^ Tg(UBC-PA-GFP)1Mnz/J	Victora et al., 2010	Jax. 022486
Mouse: Aicda-CreERT2: B6.129P2-*Aicda*^*tm1.1(cre/ERT2)Crey*^/J	Dogan et al., 2009	Jax. 033897
Mouse: cMyc-GFP: B6;129-*Myc*^*tm1Slek*^/J	Huang et al., 2008	Jax. 021935
Mouse: Eu22-Bcl2 Tg: C.Cg-Tg(BCL2)22Wehi/J	Strasser et al., 1991	Jax. 002318
Oligonucleotides		
Single-cell BCR sequencing primers	Mesin et al., 2020. IDT.	N/A
Recombinant DNA		
Fab expression vectors	Tas et al., 2016	N/A
Software and algorithms		
Flowjo	https://www.flowjo.com/	RRID:SCR_008520
Graphpad prism	https://www.graphpad.com/ RRID:SCR_002798 scientific-software/prism/	RRID:SCR_002798
Adobe Illustrator	http://www.adobe.com/products/illustrator.html	RRID:SCR_010279
GCTree	DeWitt et al., 2018	N/A
PANDAseq	https://github.com/neufeld/pandaseq	N/A
Cell Ranger	https://support.10xgenomics.com/single-cell-vdj/software/downloads/latest	RRID:SCR_017344
Biacore Insight Evaluation Software	https://www.cytivalifesciences.com/en/us/support/software/biacore-downloads	N/A
SpadeR	https://chao.shinyapps.io/SpadeR/	N/A
R Core Team (2023)	https://www.r-project.org	N/A
Other		
Fetal Bovine Serum	GIBCO	Cat#10500 056
Sheep Red Blood Cells in Alsevers	Thermo Fisher Scientific	Cat#SR0053B
Corn oil	Sigma Aldrich	Cat#C8267-500ML
DMEM	Sigma Aldrich	Cat#D5796-500ML
HEPES	GIBCO	Cat#15630080
Penicillin and Streptomycin	Thermo Fisher Scientific	Cat#15140122
Normal mouse serum	Thermo Fisher Scientific	Cat#24-5544-94
PBS-P+ (10X)	Cytiva	Cat#28995084
RNAClean XP	Beckman Coulter	Cat#A63987
AMPure XP	Beckman Coulter	Cat#A63881
TCL Buffer	Qiagen	Cat#1031576
Opti-MEM	Gibco	Cat#31985047
Expi293 Expression Medium	Gibco	Cat#A14351-01
Taq DNA polymerase	New England Biolabs	Cat#M0273L
RNasin Plus Ribonuclease Inhibitor	Promega	Cat#N2615
Maxima H minus Reverse Transcriptase	Life Technologies	Cat#EP0753
PureCube Indigo Ni-Agarose Beads	CubeBiotech	Cat#75103

## Resource Availability

### Lead contact

Any information or request for resources and reagents should be directed to and will be fulfilled by the lead contact, Oliver Bannard (oliver.bannard@ndm.ox.ac.uk).

### Materials availability

This study did not generate new unique reagents. Certain mouse lines described here were obtained from other laboratories and thus may require a Material Transfer Agreement (MTA).

### Data and code availability

- This study did not generate any unique datasets. All data reported in this paper will be shared by the lead contact upon request.
- This paper does not report original code.
- Any information required to reanalyse the data reported in this paper is available from the lead author upon request.

## Experimental Model and Study Participant Details

### Experimental mice

SW_HEL_^14^, Rosa26-LSL-tdTom ^63^, *S1pr2*-CreERT2 ^6^, Blimp1^mVenus^ ^17^, UBOW ^27^, PAGFP ^32^, *Aicda*-CreERT2 ^33^, cMyc-GFP ^64^ and E mu bcl-2-22 ^34^ mice were described previously. C57BL/6 and B6SJL.CD45.1 mice were purchased from the University of Oxford core breeding facility. Mice were 6 weeks of age or older at the time of any experimentation, and at least 8 weeks-old when challenged with influenza. Mixes of male and female mice were used. Littermates were assigned at random to experimental and control groups, ensuring equal representation in every cohort. All mice were bred, maintained and immunised in specific pathogen–free facilities at the Biomedical Sciences Facility of the University of Oxford. All experiments were performed in accordance with a project license granted by the UK Home Office and were approved by the Institutional Animal Ethics Committee Review Board of the University of Oxford.

## Method Details

### Recombinant haemagglutinin

His-tagged trimeric HKx31 HA Y98F (a point mutation reducing sialic acid binding) was produced through stable transduction of 293T cells ^65^, purified by Ni-NTA chromatography and size-exclusion using a Superdex 200 Increase 10/300 GL (Cytiva). For FACS, recombinant HA trimer was labelled with AF647 or biotin through free amine coupling chemistry (Thermo Fisher Scientific A20186, Calbiochem 203188-25MG). Only when indicated, biotin-HA was additionally tetramerised by incubation with SA-APC or SA-BV650 (Biolegend) for 30 minutes at RT, at a 4:1 (HA:SA) ratio, followed by dialysis into PBS. For SPR measurements, both WT (Y98) and Y98F HKx31 HA were expressed, purified and biotinylated as described. SPR measurements were initially performed against Y98F HA, but weakly binding infection Fabs (K_D_>10^6^M) as well as representatives of expanded clones were then re-measured against WT HA. When the Y98F mutation was found to affect the measured affinity within a clone, it was entirely re-tested against WT HA.

### Infections, HA immunisations and fate-mapping

Mice were anesthetised by isoflurane inhalation and infected intranasally with HKx31 H3N2 Influenza A virus, or immunised subcutaneously with 3µg of recombinant HKx31 HA Y98F protein in ½ volume of AddaS03 (Invivogen), on each side of the mouse. Influenza-infected mice were monitored for weight loss. For GC fate-mapping, tamoxifen (Sigma Aldrich) was administered 3 days prior to harvest, as a single intraperitoneal dose of 2mg per mouse, in corn oil with 10% ethanol. To trap PCs in LNs, FTY720 (Selleckchem) was administered intraperitoneally at 3µg/g of body weight 2 days and 1 day before harvest.

### SW_HEL_ experiments

10^5^ SW_HEL_ *S1pr2*-CreERT2 Rosa26-LSL-tdTom (with or without Blimp1^mVenus^) B cells were adoptively transferred by intravenous injection into WT C57BL/6 or B6SJL.CD45.1 recipients 1-4 days prior to intraperitoneal immunisation with HEL^3X^-conjugated SRBCs ^15^, supplemented with 10µg/mL LPS (Sigma Aldrich). Negative control mice for HEL^3X^ staining similarly received transferred SW_HEL_ B cells, but also 10^5^ OVA-specific OTII T cells, and were immunised intraperitoneally with 50µg of HEL-OVA in Sigma Adjuvant System (Sigma Aldrich) ^66^. For the detection of HEL^3X^-binding cells by flow cytometry, HEL^3X^ was conjugated to AF647 through free amine coupling chemistry (Thermo Fisher Scientific, A20186). Recombinant 6xHis-tagged HEL^3X^ protein was expressed in CHO cells and purified over a NiNTA column ^45^. WT HEL was purchased from Sigma Aldrich.

### Flow cytometry and cell sorting

Mediastinal LNs for infections or inguinal LNs for immunisations were harvested on ice in harvest media (DMEM + 1% HEPES + 1% FCS + 1% Pen/Strep), mechanically dissociated into single-cell suspensions, and washed once in harvest media prior to staining. In general, both inguinal LNs were harvested and pooled prior to staining, unless used for sorts or otherwise stated, when LNs were stained individually. Cells were first incubated with an Fc block solution (CD16/32, 10 minutes on ice) and then with fluorescently-labelled antibodies and HA probe (35 minutes on ice), both solutions prepared in FB (PBS + 1% FCS + 0.5mM EDTA). Cells were passed through 100µm cell strainers before passing on BD LSR Fortessa X20, BD LSR II, BD Aria III or BD Fusion. For intracellular staining (IRF4 or EdU), samples were fixed for 30 mins on ice with BD fixation reagent (BD Biosciences) and then permeabilised overnight at 4°C in FoxP3 permeabilisation buffer (Thermo Fisher Scientific). For EdU staining, the manufacturer’s instructions were followed (FlowPlus kit, ThermoFisher Scientific). Dump gates included Abs against CD4, CD8, TER-119 and F4/80. All flow cytometry analysis was performed with FlowJo v10 software.

### Photoconversion of individual GCs

Following the protocol thoroughly described by Jacobsen and Victora ^67^: whole LNs were carefully cleaned of fat and mounted intact on an ice-cold block under a 20X pan-apochromat objective on a Zeiss LSR780 two-photon microscope. Using either a MaiTai or Insight laser, light at 920nm was used to locate tdTom^+^ GCs without converting them. Light at 830nm was then used for photoconverting a single GC, using the lowest laser power yielding detectable conversion (the laser power required varies with sample nature and depth and should be optimised prior to GC photoconversion).

### Single-cell antibody sequencing

Plate-based method (as described in ^68^): GC B cells and/or PCs were individually sorted into separate wells of 96-well PCR plates, containing TCL buffer + 1% 2-mercaptoethanol. Following an SPRI bead clean-up, single cell RNA was reverse-transcribed to cDNA using Maxima H-Reverse Transcriptase and poly(dT) oligos. The cDNA was first subjected to PCRs to amplify separately HC VDJ and Kappa VJ (when needed) rearrangements. For the HC, a degenerate forward primer specific for the start of the VH and isotype-specific reverse primers were used; for the LC, forward primers specific for the start of the VK and a CK-specific reverse primer were used, followed by a nested PCR for further amplification. Another PCR incorporated plate-, row- and column-specific indexes. A final PCR incorporated adaptors for Illumina sequencing, and products were subsequently pooled by plate. After SPRI beads clean-up (with a beads-to-DNA ratio of 0.6), the PCR products were sequenced using an Illumina Miseq kit Nano v2, 500 cycles.

Droplet-based method (mice #3-6): A unique hashing antibody per mouse was included in the FACS staining procedure to later deconvolute samples. 2500 GC B cells and all recent PCs in each sample were two-way sorted into DMEM + 10% FCS. GC and PC samples from 4 mice were pooled by cell type and pelleted before being resuspended in 38uL of PBS and loaded, following the manufacturer’s protocol, in two separate 10X reactions on a Chromium Next GEM Chip K, using the Next GEM Single Cell 5’ Kit. Only VDJ libraries were prepared (no gene expression RNA-seq), using the 10X Genomics Chromium Single Cell Mouse BCR Amplification Kit, the 5’ Feature Barcode Kit and the Library Construction Kit. Libraries were sequenced using a NextSeq 500/550 Mid Output Kit v2.5 (150 cycles). Analysis was performed using Cell Ranger v6.1.1.

### Clonal and intraclonal analyses

PANDAseq ^69^, was used to assemble paired-end sequences before processing using a custom perl script. The script was used to: 1) identify barcodes in the assembled sequences and thereby identify original plate/well, 2) trim and filter the assembled sequences, 3) count unique sequences at each plate/well, 4) produce a final report identifying the majority sequence at each plate/well. IgH and IgK (when available) sequences were aligned to the IMGT database in order to determine the VDJ or VJ rearrangements for every cell. Clonal lineages were defined as groups of cells sharing HC V, J segments, CDR3 length and 75% or higher nucleotide identity in the CDR3 ^68^. When feasible, LC sequences were used to confirm/adjust clonal lineage assignments. Within clonal lineages, phylogenetic trees were established using GCTree ^25^, and HC VDJ sequences. Inferring UCA sequences was done by reverting somatic mutations in V and J segments away from V(D)J junctions.

### Cloning, Fab expression and affinity measurements

HC and LC variable region sequences were cloned commercially by Twist Bioscience into human HC Fab (His-tagged) and Kappa backbones ^8^. Fabs were expressed in Expi293 cells, using the ExpiFectamine 293 Transfection Kit (Gibco), and were subsequently purified using PureCube His Affinity Agarose (CubeBiotech). Very infrequently, Fabs were produced from cells for which the LC was not recovered; in such instances, the nearest available LC from other clonal members was used. For SPR measurements, 10mL cell cultures were performed, typically yielding ∼0.5-3mg of each Fab. Purified Fabs were concentrated and dialysed into PBS (to a dialysis factor of ∼6×10^7^) using 10kDa centricons (Merck). The molecular mass and extinction coefficient was theoretically estimated for each Fab, using the ExPASy ProtParam tool, and subsequently used to determine Fab molar concentrations from OD_280nm_ measurements. SPR measurements were done via single-cycle kinetics on a Biacore 8K instrument, using the Biotin CAPture kit and following manufacturer’s instructions (Cytiva). This DNA hybridisation-based immobilisation approach had the advantage of allowing chip regeneration and was adopted because we found the trimeric antigen’s native structure to be unstable under typical regeneration conditions. The combination of this approach with small cultures and single cycle kinetics facilitated moderate-throughput single-measurement K_D_ screening. Typically, ∼200-300 response units of biotinylated HA were immobilised onto the CAP sensor chip, and Fab was subsequently injected at increasing concentrations (in 1X PBS-P+, Cytiva), spanning roughly ∼9nM to ∼10µM. High-affinity Fabs were re-assessed using lower analyte concentrations. Association time: 120s, dissociation time: 600s, analyte flow rate: 30uL/min. Blank- and reference-subtracted traces were fitted with a 1:1 kinetic binding model, using the Biacore Insight Evaluation Software (Cytiva). When the kinetics rate constants were beyond the limit of the instrument, affinity measurements reported represent a steady-state analysis. K_D_ measurements which were above the maximal analyte concentration of 10µM, but for which the R_Max_ was still precisely inferred during steady-state analysis, are presented in figures in a shaded area to illustrate their lower accuracy. N.D. denotes Fabs for which R_Max_ values could not be reasonably determined, and even approximate K_D_s could not be estimated. However, N.D. Fabs did detectably bind rHA by SPR and/or multivalent surface IgM assays ^28^. In brief, surface IgM assays involved variable region heavy genes being cloned into a membrane IgM vector and co-transfected with light chains into Expi293 cells. Transfected cells were stained 3-4 days later with HA(Y98F)-AF647, human IgM-PE and human Igk-FITC. HA staining was determined relative to negative control variable region genes.

## Quantification and Statistical Analyses

The Chao1 index for clonal richness was performed using SpadeR (see Key Resources Table). Graphs and statistical tests were done using Prism v8.2.1. Statistical test information, statistical significance values, number of replicates and number of mice per group are indicated in the figure legends.

*In silico* simulations; 10,000 simulations were run where in each the observed GC cellular distribution was randomly sampled N times, where N equals the observed number of PCs, or number of PC nodes, as indicated in figure legends. We report empirical 95% confidence intervals over the 10,000 simulations for the 20 largest clone types as well as the observed values. If an association existed between GC clonal abundance and likelihood for PC differentiation, then we would expect to see a positive correlation between GC clonal size and observed PC number or node results as a percentile relative to the simulated results (i.e., where the observed data sits within expected distributions derived from simulations). This was tested by removing all clones where 0 cells were detected in GC or tdTom^+^ PC compartments, then establishing the Spearman’s Rho between abundance in GC and percentile of observed for tdTom+ PC numbers or node numbers within simulated distributions. p values report the probability that a positive correlation exists. Simulations were performed in R (R Core Team, 2023, see Key Resources Table).

## Supplementary Material

Fig S1 The association between affinity and plasma cell differentiation in a clonally restricted setting, related to Figure 1 A-C. Experimental setup as in figure 1A.
A. Representative FACS showing the Blimp1^mVenus^-based gating scheme used to identify tdTom+ PCs on d7 or d8 post-immunisation.
B. Representative FACS showing the TACI-based gating scheme used to identify tdTom+ PCs on d7 or d8 post-immunisation.
C. Percent of SWHEL among GC B cells (GL7^+^ or IgD_low_, GL7^+^) from experiments where necessary markers to identify host GC B cells were included.
D. Representative FACS showing no HEL^3X^-binding by S1pr^2Tdtom+^ SW_HEL_ GC B cells and PCs on d7 post-HEL^wt^-OVA immunisation.
E. Experimental setup as in Figure 1D. Proportion of c-Myc-GFP^+^ cells among c-Myc^gfp/wt^ SW_HEL_ GC B cells on d7 or d8 following immunisation. Each symbol is a mouse. Every timepoint is pooled from 2 experiments, each with 2-5 mice. Negative controls include both GFP- (WT) SW_HEL_ GC B cells and recipient (WT polyclonal) GC B cells. Two-tailed P value from Mann Whitney test: ****p<0.0001.

Fig S2 Plasma cell development in GCs during influenza infection, related to Figure 2 Experimental setup as in Figure 2A.
A. Surface antibody light chain (Igk) expression by GC (B220^+^ CD95^+^ tdTom^+^) and tdTom^+^ PCs (Dump-, Blimp1^+^ CD138^+^).
B. Antibody isotypes expressed by GC and tdTom^+^ PCs from sequencing results. Wilcoxon matched-pairs signed rank test: n.s. p≥;0.05, *p<0.05.
C. *Ighv* somatic mutation loads for tdTom^+^ GC B cells and PCs from mice of Figure 2D.
D. Estimated number of clonal lineages using the Chao1 index. Bars indicate 95% C.I.
E. *In silico* simulations of expected clonal PC population sizes based on random picking from GC clonal distributions, assuming each observed PC in Figure 2D represents a unique selection event, repeated 10,000x to establish 95% confidence intervals (bars). Comparisons to observed results (circles) are shown for the 20 largest GC clones.
F. Similar simulations as in E but considering each unique PC sequence (somatic variant) to represent a selection event (i.e., the sampling number).
Spearman’s Rho tests a correlation between GC abundance and the observed data percentile in relation to simulated ranges across clones. P values report the probability of a positive correlation.

Fig S3 PC differentiation traced to GC maturation pathway and PC node generation, related to Figure 3 A. Phylogenetic maturation trees for the indicated GC clones of Ms#5 from figure 2D, and observed population sizes for tdTom^+^ PCs. Coloured nodes indicate sequences observed in both GC and PC compartments. Numbers and node sizes indicate number of observed cells with identical HC VDJ sequences. Number-less nodes are inferred but not observed, arrows indicate where PC differentiation maps to them.
B. Observed node sizes for tdTom^+^ PCs in the mice of Figure 2D (numbers of cells observed with identical HC VDJ sequences).

Fig S4 Plasma cell differentiation during a late-stage antiviral response, related to Figure 4 A-D. S1pr2^tdTom^ Blimp1^mVenus^ medLNs were harvested on d21 post-infection, following an injection of tamoxifen on d18 and FTY720 injections on d19 and d20. HA^+^ tdTom^+^ GC B cells and PCs were sorted and sequenced similar to d14 mice from Figure 2.
A. Pie charts showing the distribution into clonal lineages of tdTom^+^ GC B cells and PCs. Each slice is a distinct clone. Coloured slices indicate lineages shared between the GC and PC compartments of a given mouse. Numbers indicate: “Nb of clonal lineages/Nb of cells sequenced”.
B. Phylogenetic maturation trees for the indicated clones, and observed population sizes for tdTom^+^ PCs. Coloured nodes indicate sequences observed in both GC and PC compartments. Numbers and node sizes indicate number of observed cells with identical HC VDJ sequences. Number-less nodes are inferred but not observed, arrows indicate where PC differentiation maps to them.
C. Observed node sizes for tdTom^+^ PCs (numbers of cells observed with identical HC VDJ sequences).
D. *Ighv* somatic mutation loads of GC B cells and PCs from major clones across all 3 analysed mice.

Fig S5 GC plasma cell output after subunit immunisation, related to Figure 6 A. GC size-normalised PC output (number of tdTom^+^ PCs per 1000 tdTom^+^ GC B cells) in *Aicda*-CreERT2 Rosa26-LSL-tdTom mice on d14 and d21 post-influenza infection, or on d14 post-immunisation s.c. with HA/AddaS03. Each symbol is a mouse. Each condition is pooled from 2 experiments, each with 3-4 mice. Recent PCs were gated as Dump-IgD-CD138^+^tdTom^+^.
B. Experimental setup as in Figure 6A, except using CGG/Alum s.c. immunisation. GC size683 normalised PC output in iLNs on d14 post-challenge. Compare values to Figure 6B. Data pooled from two experiments, each with 3-4 mice.
C. The same experimental scheme as in Figure 6A, but with EdU injections 1 hour before analysis on day 14. Proportion of EdU^+^ cells among the indicated cell type. Each symbol is a mouse. Data pooled from 2-3 experiments, each with 2-3 mice per condition. Grey symbols from infection are shown for comparison but are the same data as in Figure 3F.
D. Proportion of tdTom^+^ among GC B cells 30 hours after tamoxifen treatment, in the mice from Figure 6E. Each symbol is a mouse.
E. Continuation of Figure 6F, showing observed tdTom^+^ GC B cell and PC clonality from additional mice. Each pie is one iLN from one mouse, except 12A (Fig. 6F) and 12B which represent both iLNs from a single animal.
F. *In silico* simulations of expected PC node numbers per clone, based on random sampling from GC clonal distributions using the total observed node number for each mouse as the sampling N, repeated 10,000x to establish 95% confidence intervals (bars). Comparisons to observed results (circles) are shown for the 20 largest GC clones.
G. Additional examples of phylogenetic maturation trees and tdTom^+^ PC nodes from day 14 post- HA/AddaS03 immunisation (extension of Figure 6G). Coloured nodes indicate sequences observed in both GC and PC compartments. Numbers and node sizes indicate number of observed cells with identical HC VDJ sequences. Number-less nodes are inferred but not observed, arrows indicate where PC differentiation maps to them.
H. Observed node sizes for tdTom^+^ PCs (numbers of cells observed with identical HC VDJ sequences) for each mouse. Total observed node number is indicated. I-J. Draining iLNs from S1pr2^tdTom^ Bcl2^Tg^ and S1pr2^tdTom^ littermate controls from d14 or d15 following s.c. HA/AddaS03 immunisation and 3d-tamoxifen treatments (with FTY720 treatments). Data pooled from 3 experiments, each with 1-3 mice per condition.
I. Total PC numbers per LN. Each symbol is a mean from 2 LNs per animal.
J. GC size-normalised PC output in S1pr2^tdTom^ Bcl2^Tg^ and S1pr2^tdTom^ littermate controls. Unpaired t tests with Welch’s correction (A , C, D, I, J): n.s. p≥0.05, * p<0.05, **p<0.01, ****p<0.0001. Spearman’s Rho tests a correlation between GC abundance and the observed data percentile in relation to simulated ranges across clones. P values report the probability of a positive correlation.
