# Supplementary figures and images for: Germinal centers output clonally diverse plasma cell populations expressing high and low affinity antibodies
Source: Cell. Author manuscript; Available in PMC 2025 Feb 16. (PMC7617393; doi:10.1016/j.cell.2023.10.022)

# A

## Blimp1-Venus gating scheme

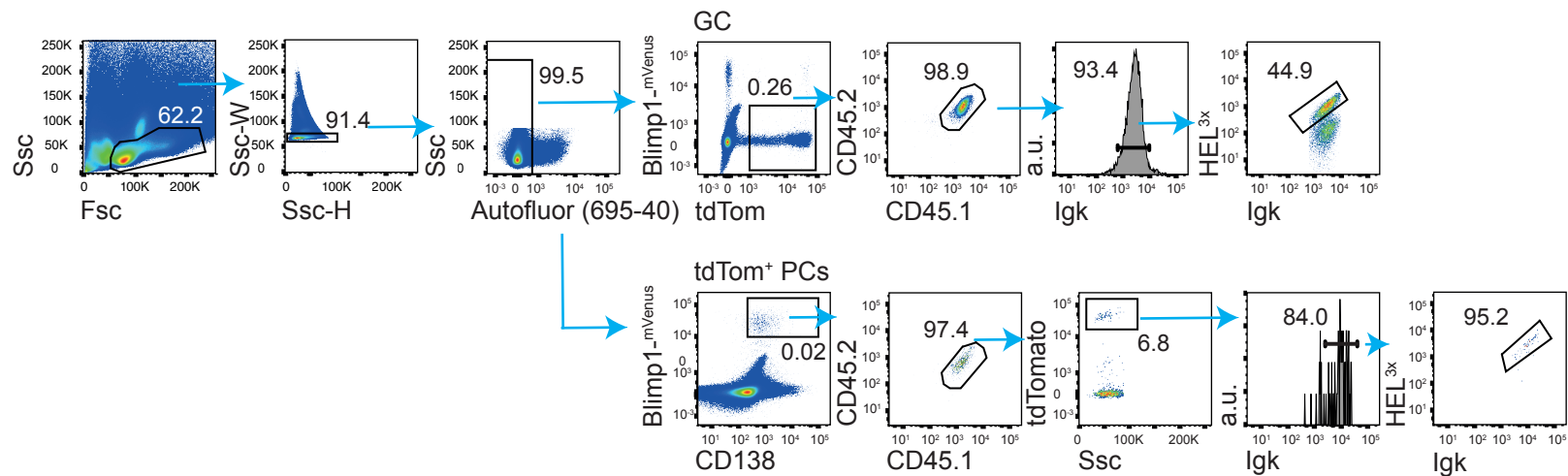

# B

## TACI gating scheme

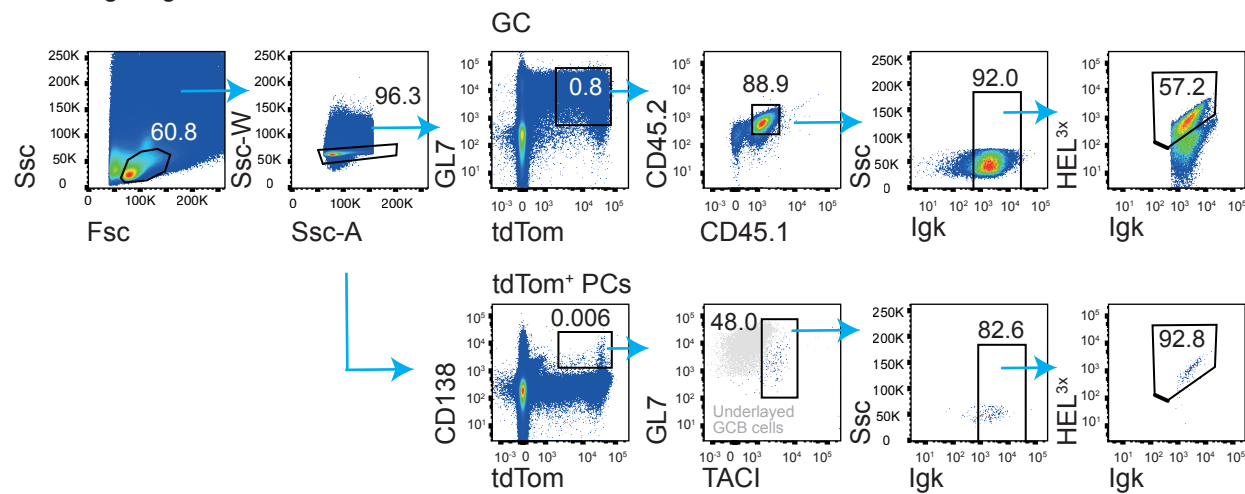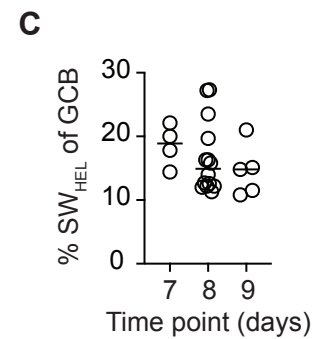

# D

## HEL<sup>wt</sup>-SRBCs immunized controls

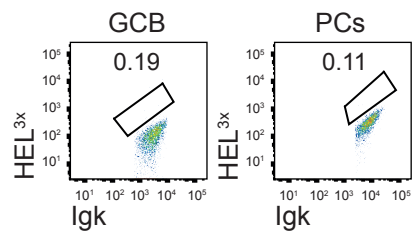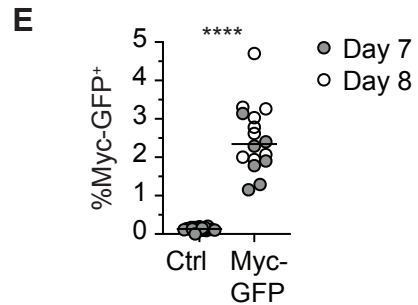

Supplement: Fig S1 [file EMS203264-supplement-Fig_S1.pdf]

**A**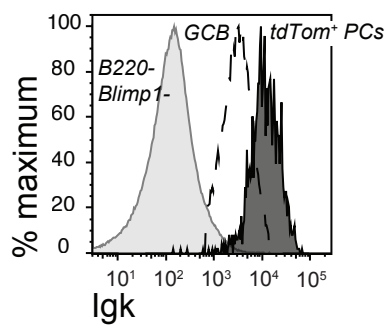**B**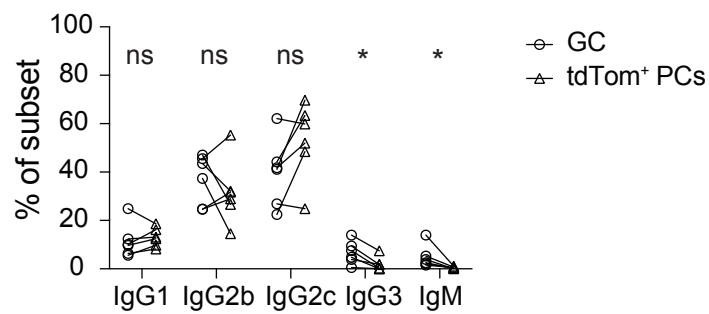**C**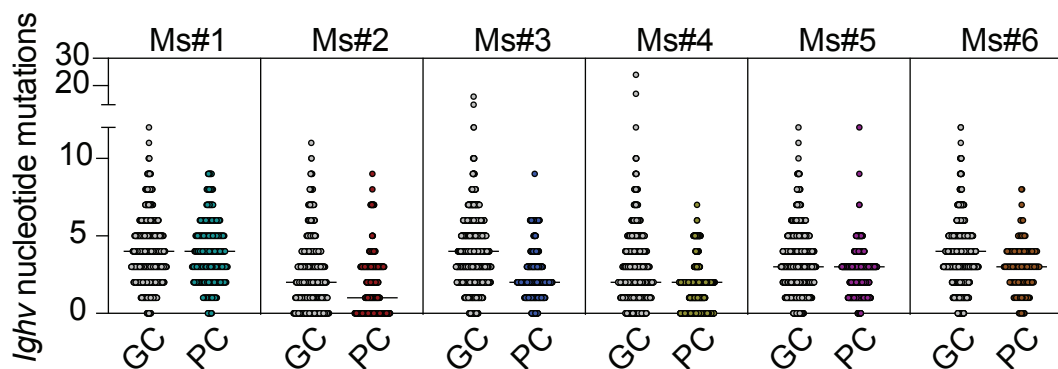**D**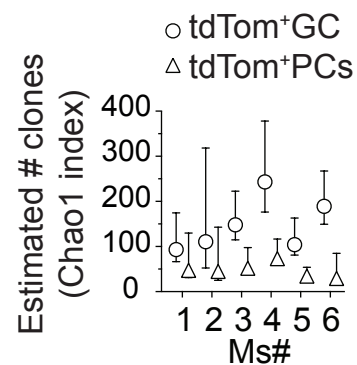**E**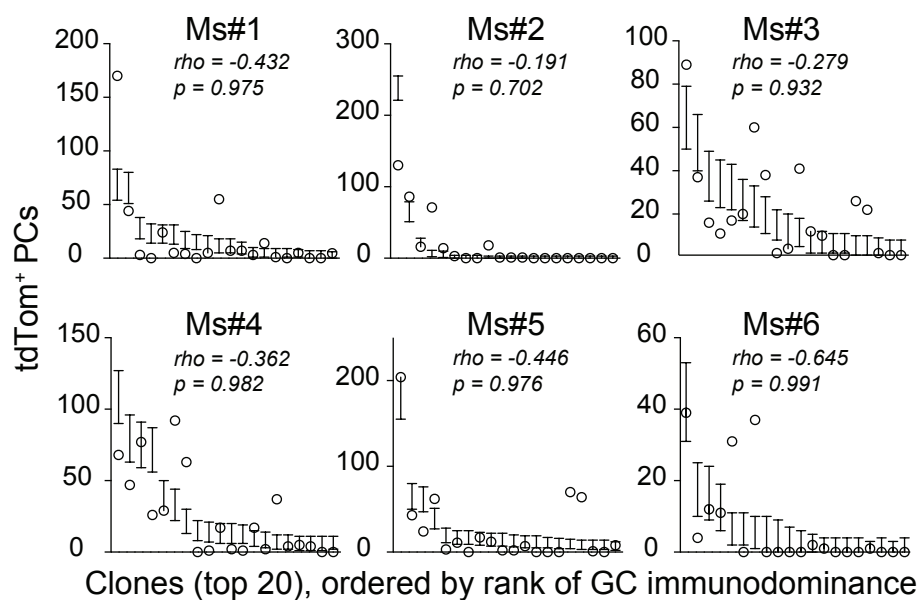**F**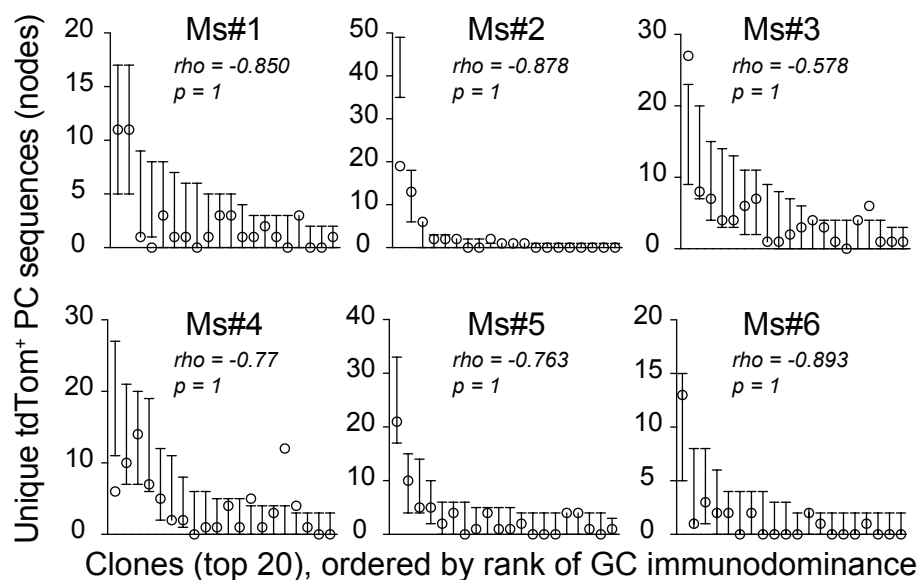

Supplement: Fig S2 [file EMS203264-supplement-Fig_S2.pdf]

## Day 21 infection

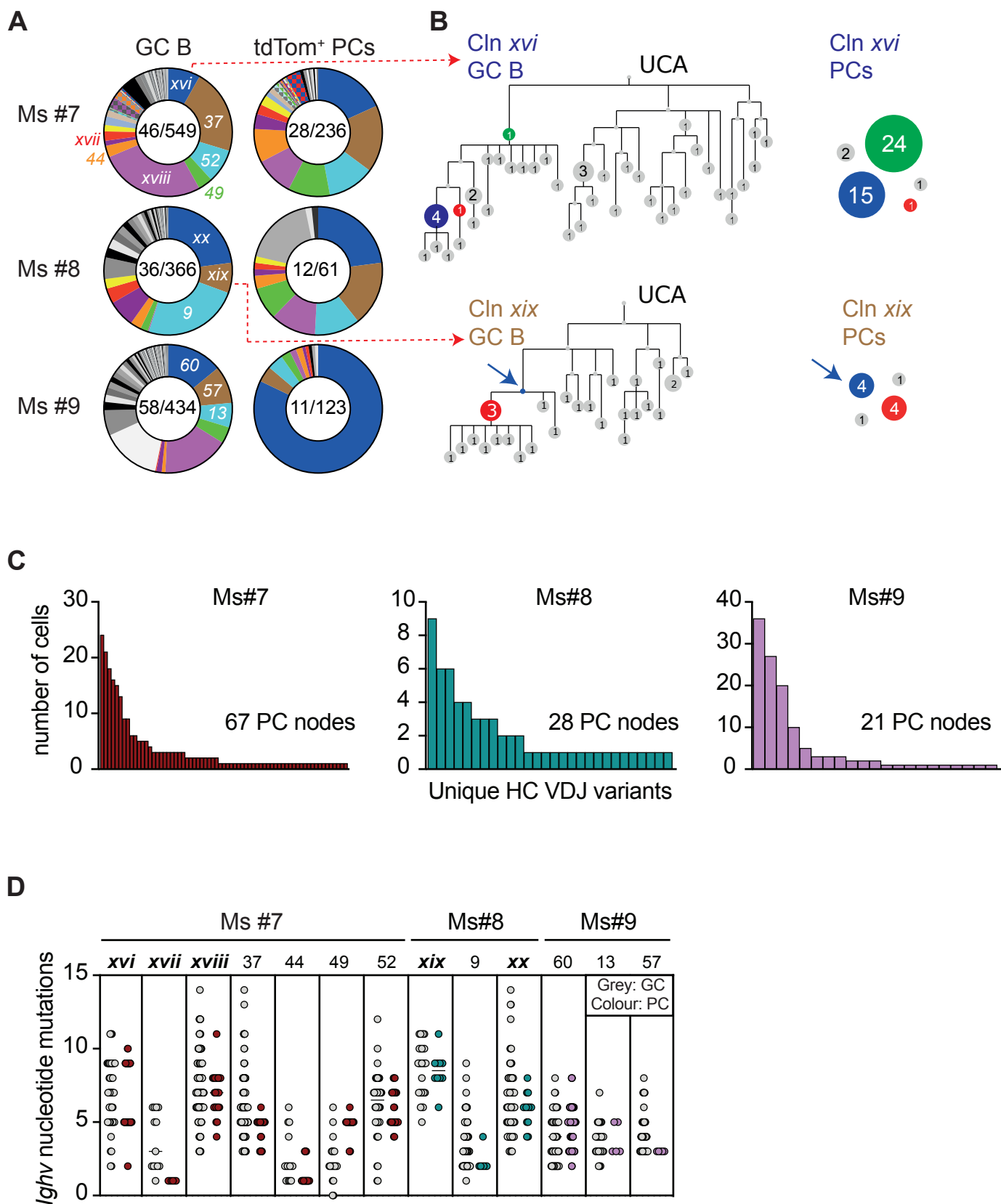

Supplement: Fig S4 [file EMS203264-supplement-Fig_S4.pdf]

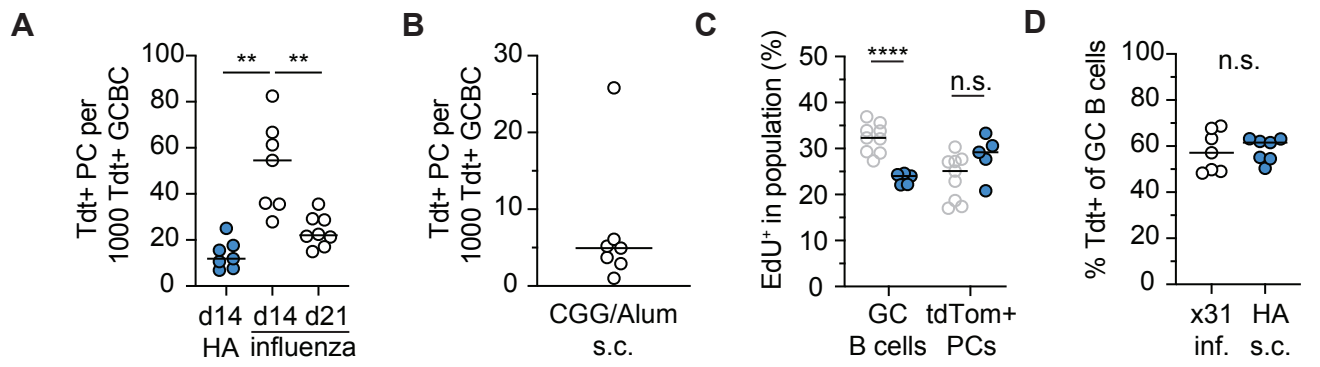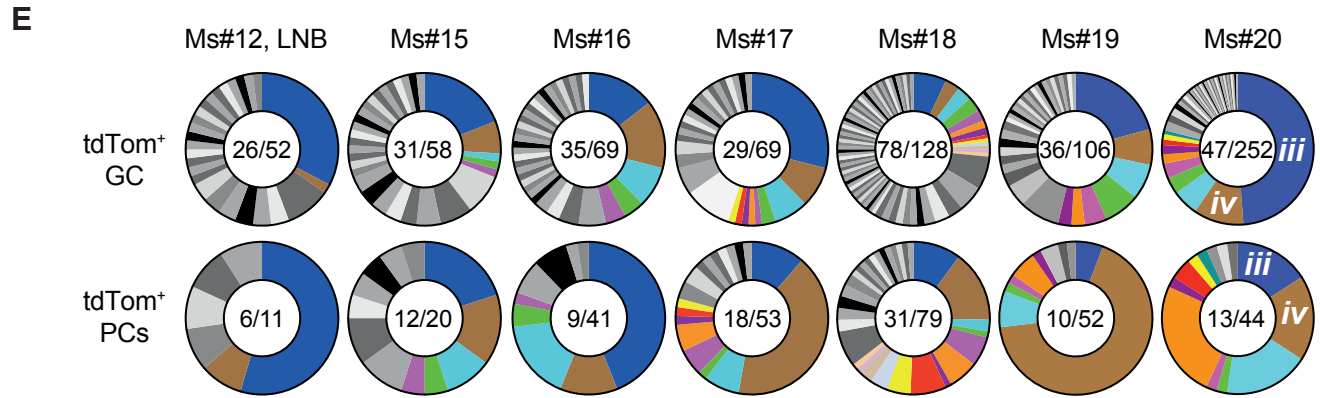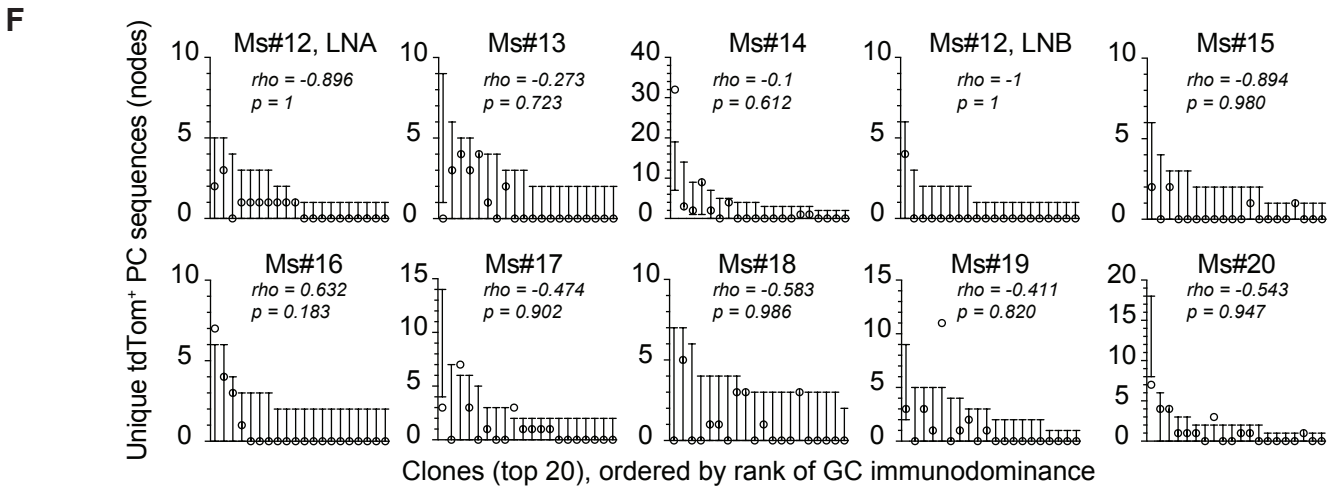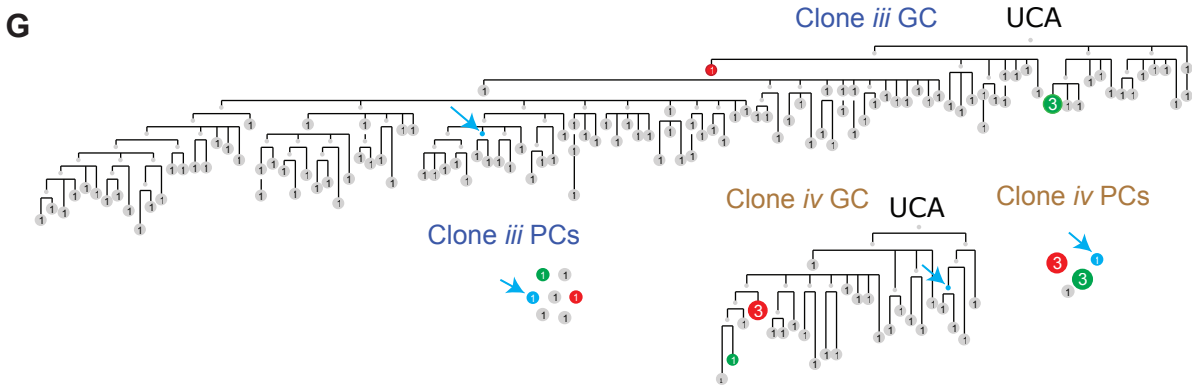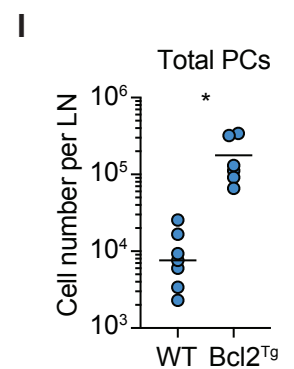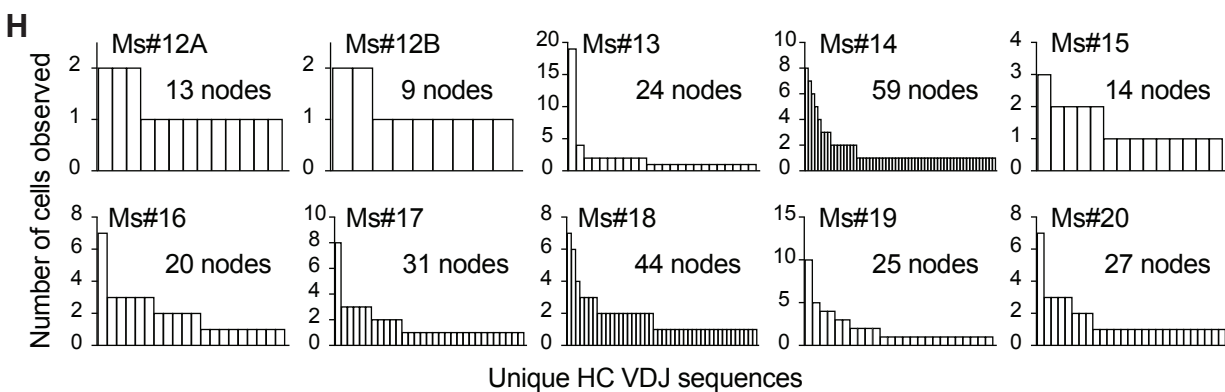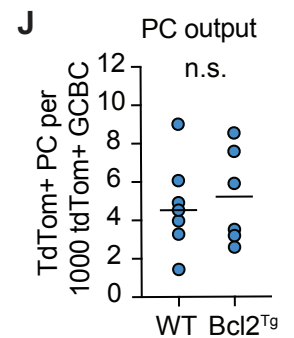

Supplement: Fig S5 [file EMS203264-supplement-Fig_S5.pdf]
