# Supplementary material for: Germinal centers output clonally diverse plasma cell populations expressing high and low affinity antibodies
Source: Cell. Author manuscript; Available in PMC 2025 Feb 16. (PMC7617393; doi:10.1016/j.cell.2023.10.022)
Supplement: Fig S3 [file EMS203264-supplement-Fig_S3.pdf]

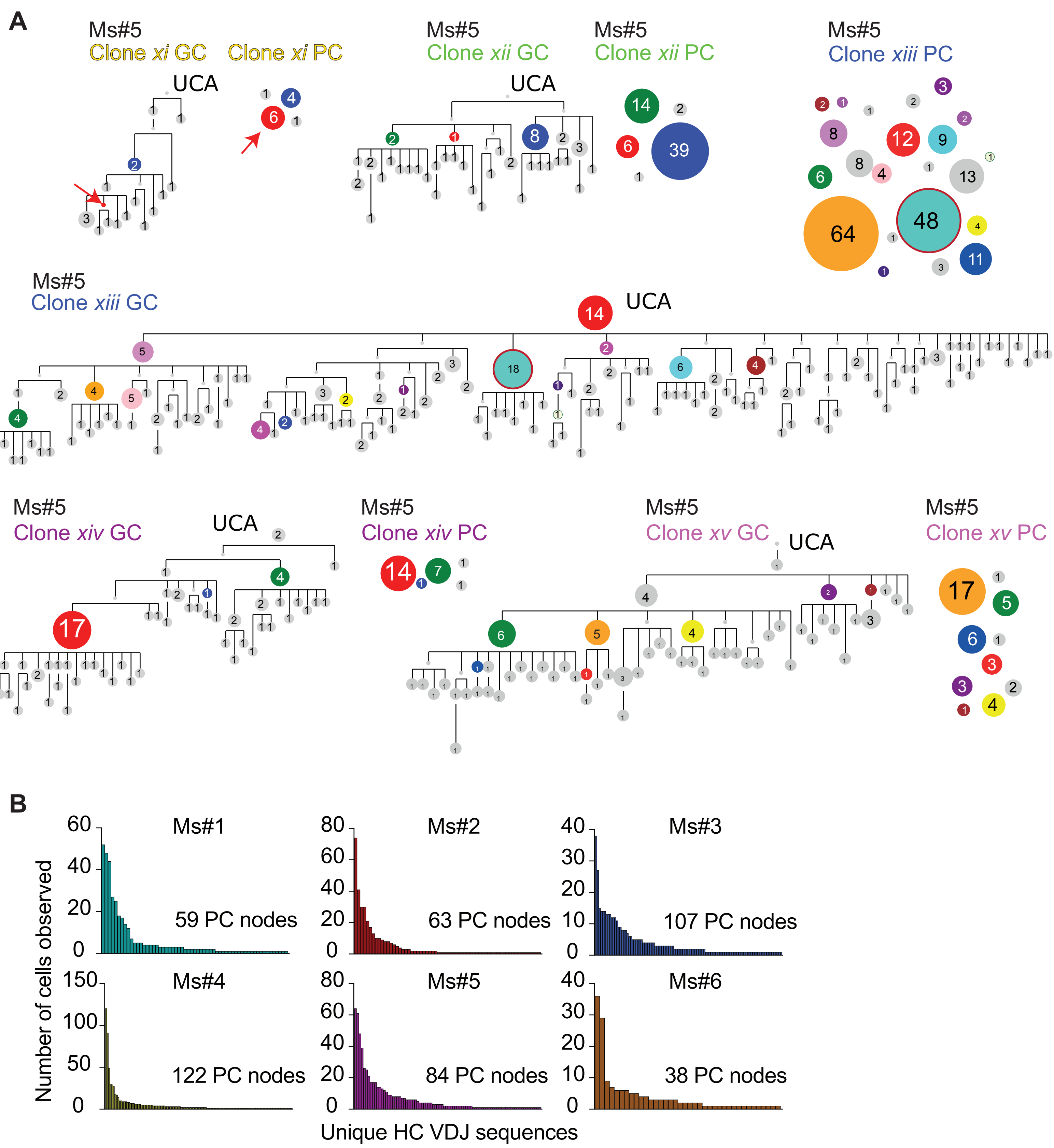
A
Ms#5
Clone xi GC
Clone xi PC
UCA
Ms#5
Clone xii GC
Ms#5
Clone xii PC
UCA
Ms#5
Clone xiii PC
Ms#5
Clone xiii GC
UCA
Ms#5
Clone xiv GC
UCA
Ms#5
Clone xiv PC
Ms#5
Clone xv GC
UCA
Ms#5
Clone xv PC
B
Number of cells observed
Ms#1
59 PC nodes
Ms#2
63 PC nodes
Ms#3
107 PC nodes
Ms#4
122 PC nodes
Ms#5
84 PC nodes
Ms#6
38 PC nodes
Unique HC VDJ sequences
